# Elevational and Seasonal Patterns of Plant–Hummingbird Interactions in a High Tropical Mountain

**DOI:** 10.1002/ece3.70469

**Published:** 2024-10-24

**Authors:** Eugenia M. Sentíes‐Aguilar, Silvana Martén‐Rodríguez, Guillermo Huerta‐Ramos, Sergio Díaz‐Infante, Gabriel López‐Segoviano, Armando Aguirre‐Jaimes, Mauricio Quesada‐Avendaño, Jorge Cortés‐Flores, María del Coro Arizmendi

**Affiliations:** ^1^ Laboratorio Nacional de Análisis y Síntesis Ecológica, Escuela Nacional de Estudios Superiores Universidad Nacional Autónoma de México (UNAM) Morelia Michoacán Mexico; ^2^ Posgrado en Ciencias Biológicas Universidad Nacional Autónoma de México Morelia Michoacán Mexico; ^3^ Facultad de Estudios Superiores Iztacala, Lab. de Biodiversidad y Cambio Global Universidad Nacional Autónoma de México Tlalnepantla Estado de México Mexico; ^4^ Instituto de Ecología, A. C., Red de Interacciones Multitróficas Xalapa Veracruz Mexico; ^5^ Departamento de Ecología Tropical, Campus Ciencias Biológicas y Agropecuarias Universidad Autónoma de Yucatán Mérida Mexico; ^6^ Jardín Botánico, Instituto de Biología, Sede Tlaxcala Universidad Nacional Autónoma de México Santa Cruz Tlaxcala Mexico; ^7^ Facultad de Estudios Superiores Iztacala, UPIBRO, Lab. de Ecología Universidad Nacional Autónoma de México Tlalnepantla Estado de México Mexico

**Keywords:** diversity, elevation gradient, hummingbirds, Nevado de Colima, phenology, pollination networks

## Abstract

Tropical mountain ecosystems harbor diverse biological communities, making them valuable models for exploring the factors that shape ecological interactions along environmental gradients. We investigated the spatial and temporal drivers of plant–hummingbird interaction networks across three forest types (pine‐oak, fir, and subalpine) along a tropical high mountain gradient in western Mexico (2400 to 3700 m.a.s.l.). We measured species abundance, diversity, morphology, and interaction frequencies. Plant diversity metrics significantly declined in the highest elevation subalpine forest, whereas hummingbird diversity remained consistent across elevations. Interaction networks were similarly nested across elevations, but they were more specialized in the subalpine forest, where lower plant species richness and higher floral abundance led to greater resource partitioning among hummingbirds. Plant–hummingbird networks were larger and less specialized during the dry season, driven by greater species diversity and abundance. Species turnover explained network variation along the elevational gradient, while interaction rewiring and the arrival of migratory hummingbirds explained changes between seasons. Phenological overlap was the most important driver of the observed variation in interaction frequencies across elevations and seasons. Flower abundance had a minor influence on interaction frequencies at low‐ and mid‐elevation networks, and hummingbird abundance was significant for dry‐ and rainy‐season networks. Morphological matching was significant in the low‐elevation forest and in the dry season. Plant phylogenetic relatedness had negligible effects on interaction patterns, but hummingbird phylogeny influenced feeding preferences in high‐elevation and rainy‐season networks. Our findings highlight the role of species turnover, interaction rewiring, and phenological overlap in structuring plant–hummingbird networks, with specific effects of abundance, morphology, and phylogeny varying with elevation and season. High‐elevation ecosystems play a crucial role as reservoirs of floral resources for both resident and migratory hummingbirds during resource‐scarce periods, emphasizing their importance in maintaining biodiversity in tropical mountain gradients.

## Graphical abstract

This study examined the patterns and potential drivers of diversity and spatiotemporal dynamics of plant–hummingbird interactions across Mexico's high tropical elevational gradient. Results revealed the importance of species turnover, interaction rewiring, and phenological overlap in network structuring, with smaller roles of abundance, morphology, and phylogeny varying by elevation and season. These findings highlight the importance of conserving tropical mountain ecosystems, which support critical ecological interactions.

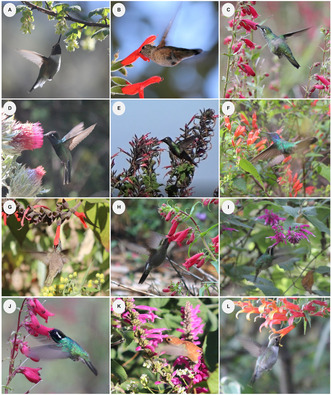

## 1 Introduction

Plant–pollinator interactions play an essential role in the assembly of terrestrial communities by directly contributing to the reproduction of numerous angiosperm species and the survival of many animal species (Mayer et al. 2011). These mutualistic interactions vary along environmental gradients based on the richness, abundance, composition, and phenology of the interacting species (Arroyo, Primack, and Armesto 1982; Dalsgaard et al. 2009; Morellato et al. 2016). High mountain regions encompass steep topographical and climatic environmental gradients that provide excellent study systems for assessing the factors underlying the patterns of diversity and structuring of plant–pollinator interactions (Beck and Chey 2008; Kessler and Kluge 2008; Fischer, Blaschke, and Bässler 2011). In Neotropical montane regions, plant–hummingbird interactions are crucial in the maintenance of understory and epiphytic plant communities (e.g., Krömer, Kessler, and Herzog 2006; Maglianesi et al. 2014; López‐Segoviano et al. 2021). Evaluating the spatiotemporal dynamics of plant–hummingbird interactions and the factors that influence their establishment provides valuable insights into the assembly of communities along mountain gradients; however, studies encompassing high mountain ecosystems along tropical elevational gradients are still limited (but see Gutiérrez, Rojas‐Nossa, and Stiles 2004; Weinstein and Graham 2017; Partida‐Lara et al. 2018).

Hummingbirds are prominent pollinators of mountain forests and the only nectar‐feeding vertebrates known to inhabit ecosystems above 4000 m.a.s.l. (Projecto‐Garcia et al. 2013; Arredondo‐Amezcua et al. 2018; Williamson et al. 2024). Their ability to thrive in high‐elevation environments is due to a range of physiological and behavioral adaptations, including genetic variants of hemoglobin proteins that enhance oxygen transport (Williamson et al. 2023), the capacity to enter torpor (i.e., a state of deep rest where metabolism drops to conserve energy during cold weather or food scarcity) (Wolf et al. 2020), and flexible feeding behavior to regulate oxygen consumption and heat or energy loss (e.g., perching while feeding rather than hovering) (Stiles 2004; Arredondo‐Amezcua et al. 2018). In montane ecosystems, hummingbirds can also synchronize their life cycle according to the spatiotemporal availability of floral resources, contributing to the reproductive success of the plant species they pollinate (Cruden 1972; Partida‐Lara et al. 2012; Zanata et al. 2017; Pelayo et al. 2019).

In tropical mountains, changes in the diversity and community composition of plants and pollinator species influence the structuring of interaction networks along elevational gradients (Du et al. 2021). Plant and animal diversity patterns vary according to the extent and scale of the gradient (Rahbek 1997, 2005; Nogués‐Bravo et al. 2008; Guo et al. 2013), as well as the humidity conditions of the mountain slope (McCain and Grytnes 2010). Linear declines and hump‐shaped diversity patterns are the most commonly reported for plants and birds along a wide range of elevational gradients (e.g., Kessler 2001; Rahbek 2005; Nogués‐Bravo et al. 2008). Specifically for hummingbirds, both patterns have also been documented (e.g., Maglianesi et al. 2015; Moreira, Falcão, and de Araújo 2020; López‐Segoviano et al. 2021). However, in high mountains, species diversity generally shows a monotonical decrease above an intermediate elevation threshold (McCain and Grytnes 2010). Mountain gradients are also characterized by a high species turnover in the plant (e.g., Cordeiro et al. 2023; Minachilis et al. 2023) and hummingbird communities (e.g., Weinstein et al. 2014; Maglianesi et al. 2015). Evaluating beta diversity of interactions sheds light on the contribution of changes in species composition versus the rewiring of species interactions as drivers of spatial and temporal variation in interacting networks (e.g., Gómez‐Murillo and Cuartas‐Hernández 2016; Souza et al. 2021; Dzekashu et al. 2023).

Interaction network approaches enable the evaluation of factors that influence the establishment and structuring of plant–hummingbird interactions (e.g., Dalsgaard et al. 2008; Vizentin‐Bugoni et al. 2018). Through this approach, several factors have been identified as key determinants of interaction frequencies, including species abundance (a neutral process; Vázquez et al. 2007), phenological overlap (e.g., Vizentin‐Bugoni, Maruyama, and Sazima 2014; Gonzalez and Loiselle 2016; Chávez‐González et al. 2020), and morphological coupling (e.g., Maruyama et al. 2014; Weinstein and Graham 2017; Sonne et al. 2019). In addition, phylogeny can partly explain interaction frequencies, as closely related species are expected to share specific interactions more often than distantly related species (e.g., Rezende, Jordano, and Bascompte 2007; Graham et al. 2012; Martín‐González et al. 2015). In the few studies available for tropical mountains, phenological and morphological matching have emerged as the most relevant factors influencing plant–hummingbird interactions (e.g., Vizentin‐Bugoni, Maruyama, and Sazima 2014; Gonzalez and Loiselle 2016; Vitória, Vizentin‐Bugoni, and Duarte 2018; Sonne et al. 2020). At intermediate tropical latitudes, where latitudinal and elevational hummingbird migrants overlap, hummingbird migrations are also likely important drivers of temporal variation in network structuring (e.g., Contreras‐Martínez 2015; Licona‐Vera and Ornelas 2017; López‐Segoviano et al. 2018).

Elevational patterns in plant–hummingbird interactions are still underexplored in tropical mountain ecosystems. We selected a mountain gradient in Nevado de Colima, the highest peak of central‐western Mexico, located at a northern tropical latitude. This region supports high hummingbird diversity due to the coexistence of resident species, as well as latitudinal and elevational migrants, making it an essential year‐round habitat for hummingbirds (Des Granges 1979; Contreras‐Martínez 2015). To assess variation in diversity and interaction patterns along the mountain slope, we aimed to (1) determine the patterns of abundance and diversity of blooming plant and hummingbird species across the elevational gradient during the dry and rainy seasons; (2) describe the phenology of the interacting plant and hummingbird communities across the year; (3) document the variation in plant–hummingbird interaction networks across elevations and seasons; and (4) analyze the influence of abundance, phenology, morphology, and phylogeny on interaction frequencies across elevations and seasons.

Given that the study encompasses a mid to high elevational gradient and that mid‐elevations in dry‐slope mountains provide wetter and warmer conditions, we expected a decrease in species richness, abundance, and diversity with elevation (McCain and Grytnes 2010). We predicted a higher abundance and diversity of hummingbirds during the dry season, as mountains in central‐western Mexico experience a flowering peak and the arrival of migratory hummingbirds (Schondube et al. 2005). Species turnover was expected due to variation in flowering phenologies and the presence of migratory hummingbirds (Des Granges 1979; Stiles 1985; Weinstein et al. 2014; Maglianesi et al. 2015). Since higher abundance and diversity generally promote greater food partitioning, we predicted more specialized interactions at the elevation and season with the greatest richness of flowering plants and hummingbirds (Dalsgaard et al. 2011; Maglianesi et al. 2014, 2015). We expected abundance to influence interaction frequencies at the more diverse forest sites (Vázquez and Aizen 2005). However, in less diverse habitats, we anticipated that flowering phenologies and hummingbird seasonality would play a more significant role, particularly given the presence of migratory hummingbirds that rely on available floral resources (Arizmendi 2001; Maruyama et al. 2014; Vizentin‐Bugoni, Maruyama, and Sazima 2014; Gonzalez and Loiselle 2016). We expected a minor influence of morphological matching due to reduced phenotypic variation among hummingbird species in this community (Rodríguez‐Flores et al. 2019; Sonne et al. 2020). Finally, since environmental filters limit the number of lineages adapted to mountain environments (Qian, Ricklefs, and Thuiller 2021), we expected plant and hummingbird communities to be less diverse at the highest elevations, leading to a greater influence of phylogeny on interaction strengths.

## 2 Methods

### 2.1 Study Site

**FIGURE 1:**
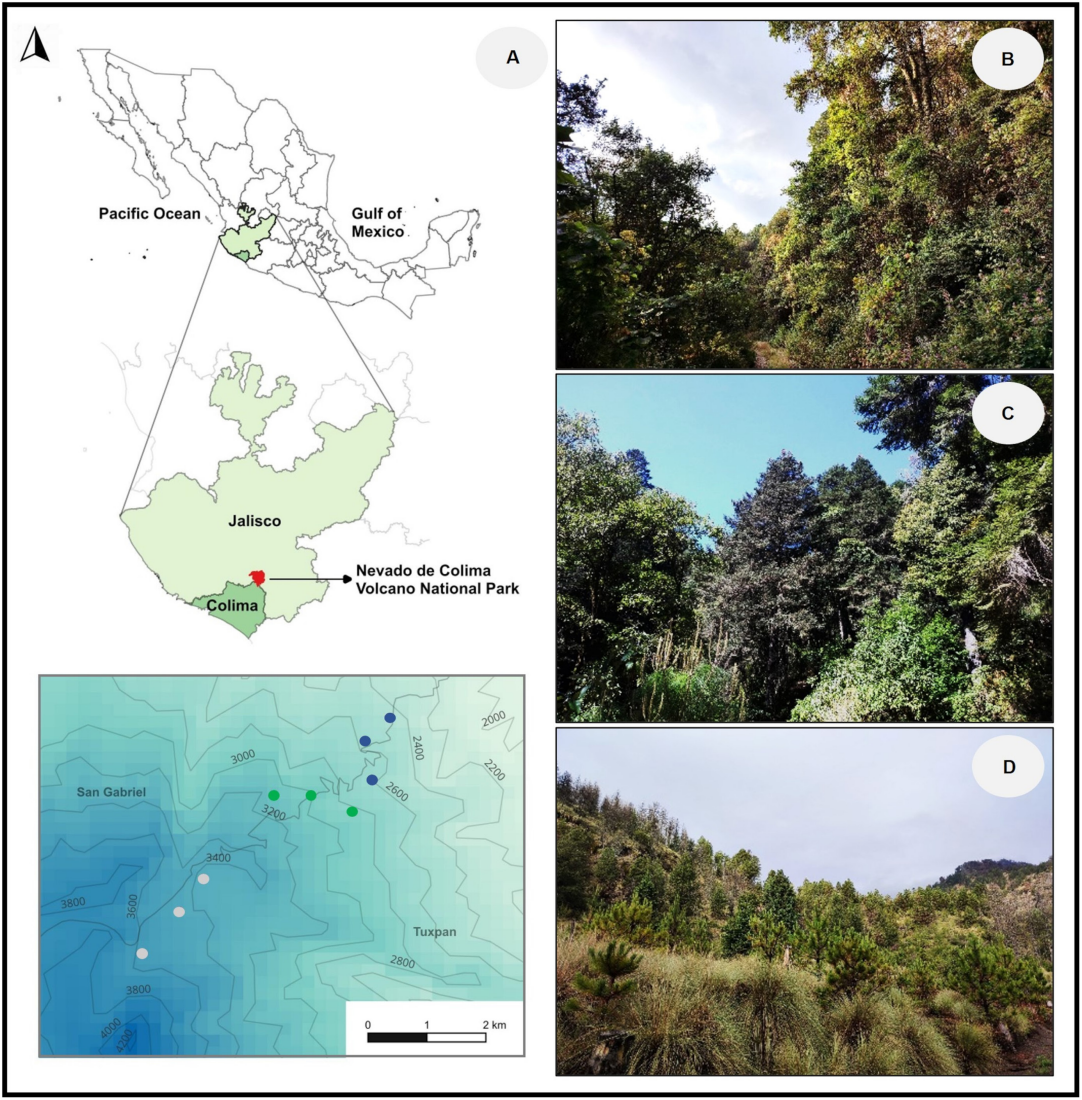
Map showing Nevado de Colima Volcano National Park, Mexico. (A) Study site area showing the sampling transects along the elevational gradient. Increasing map color intensity indicates an elevation increase (m.a.s.l.), shown on contour lines: Low site (blue dots), middle site (green dots), and high site (gray dots). Physiognomy of the different ecosystems: (B) pine‐oak forest (Low), (C) fir forest (Mid), and (D) subalpine forest (High). The map was built using the free access software QGIS v. 3.32 (QGIS Development Team 2023). Photographs by Eugenia Sentíes.

The study was conducted in Nevado de Colima Volcano National Park (peak 4270 m.a.s.l.; 19°33′45.0″ N, 103°36′31.0″ W; Figure 1), which is located at the western end of the Trans‐Mexican Volcanic Belt, in the state of Jalisco, Mexico (Figure 1A). Nevado de Colima Volcano is the highest mountain in western Mexico and its associated elevational gradient includes habitats above 2400 m with relatively little human disturbance (CONANP 2006). In ascending order, the native ecosystems found along the north‐eastern slope of the gradient include pine‐oak forest (*Pinus* spp. and *Quercus* spp.), fir forest (*Abies colimensis* [Rushforth & Narave]), *Hartweg's* pine forest (*Pinus hartwegii* Lindl., *Alnus jorullensis* [Kunth]), and the alpine grassland at the highest elevation (CONANP 2006; Cuevas‐Guzmán et al. 2011; Maarse, Verweij, and Pérez 2012). The criteria to classify the forests at Nevado de Colima corresponded to those used for montane ecosystems of the Trans‐Mexican Volcanic Belt (Leopold 1950; Miranda and Hernández 1963; Rzedowski and Huerta 1978).

We conducted our sampling at three elevations in the north face of the mountain slope between 2400 and 3700 m. The lower elevation corresponds to a pine‐oak forest, with transects located between 2400 and 2600 m (Figure 1B); at 2400 m, the mean annual temperature and precipitation are 13.4°C and 1070 mm, respectively (Sánchez‐Ramos et al. 2022). The intermediate elevation corresponds to the fir forest, which comprises a genetically differentiated population of *A. colimensis* in the Trans‐Mexican Volcanic Belt (Jaramillo‐Correa et al. 2008) with a status of endemism and protection (NOM‐059‐SEMARNAT‐2010 2019). Sampling transects in this forest type were established between 2800 and 3100 m (Figure 1C); at 2900 m, the average annual temperature and precipitation are 11.2°C and 1170 mm, respectively (Sánchez‐Ramos et al. 2022). The highest elevation corresponds to *Hartweg's* pine forest, hereafter subalpine forest, with transects located between 3400 and 3700 m (Figure 1D). At 3500 m, the mean annual temperature and precipitation are 9.3°C and 1284 mm, respectively (Sánchez‐Ramos et al. 2022). Plant and hummingbird surveys were conducted bimonthly over a two‐year period (2020–2022). We conducted six surveys during the dry winter–spring season (November, January, March) and six during the rainy season (May, July, September), across three 1‐km‐long transects established at each elevation. Hereafter, the three sampling areas will be referred to as low‐, mid‐, and high‐elevation forests; however, these are relative terms, since our sampling was restricted to the upper middle portion of the elevational gradient.

### 2.2 Plants

To determine the richness and abundance of plant species used by hummingbirds for nectar, we established six‐point counts along each transect (18 per elevation); at each point count, we established a fixed 30 m radius and documented all plant species visited within this area. We recorded the number of flowering individuals and the number of open flowers per individual. When flower density was high, the total number of flowers was estimated by extrapolating the number of flowers from a sampled plot to the total area. Plant voucher specimens (field permit number FAUT‐0366) were identified and deposited in “Herbarium Luz María Villarreal de Puga” (IBUG), Universidad de Guadalajara, and “Herbarium Graciela Calderón y Jerzy Rzedowsky” (IEB), Instituto de Ecología, A.C. (see Appendix S1 for ID voucher numbers). To assess the morphological matching between flowers and hummingbird bills, we measured floral tube length and corolla curvature in 15–30 individuals per plant species, in four to six flowers per individual.

### 2.3 Hummingbirds

To determine the abundance and richness of hummingbirds along the elevational gradient, we recorded the individuals that were visually and aurally detected for 10 min, within the 30 m radius of each point count. Point counts were separated by 200 m to minimize the risk of double‐counting individuals. Direct observations were conducted within 4 h of sunrise using binoculars (Eagle Optics Ranger ED 8 × 42). This year‐round sampling design allowed us to record both resident and migratory species. The latter includes elevational migratory species that follow resource availability, and latitudinal migratory, which travel from their northern breeding grounds to warmer latitudes during the fall–winter season.

To obtain morphological measurements (length and curvature of the exposed culmen), we captured hummingbirds with six mist nets (12 × 2.5 m with a mesh size of 24 mm), placed along the transects. The nets were open for 4 h, from 7:00 to 11:00 h, and checked every 15–20 min. One netting session per elevation was carried out during the 12 sampling periods. Bird capture was conducted under the ethical guidelines of the North American Bander's Manual for Hummingbirds (Russell, Russell, and Hill 2018). To increase the sample size for morphological traits, we added data from museum specimens measured at Alfonso Herrera Museum of Zoology (MZFC‐HE) and the National Bird Collection (CNAV), Universidad Nacional Autónoma de México, as well as the Bird Collection of Universidad Michoacana de San Nicolás de Hidalgo (CAFB‐UMSNH).

### 2.4 Plant–Hummingbird Interactions

We recorded plant–hummingbird interactions through direct observations in the same transects and sampling periods to obtain interaction frequencies. Direct observations were conducted from 7:00 to 14:00 for each plant species, for a total of 1012 observation hours. To maintain consistent sampling conditions throughout the year, we selected a schedule that avoided afternoon sampling, as this period is often rainy and misty during the rainy season. At least nine individuals of each plant species were observed for 1‐h periods on various days. To obtain pollinator visitation rates, we recorded the number of flowers observed and the number of flowers visited by each hummingbird species. Analyses were based on legitimate visits, excluding nectar theft, where hummingbirds do not contact the plant's reproductive organs (Anselmo et al. 2023). Direct observations were complemented by 1‐h video recordings (GoPro Hero 8 Black and Sony cameras) conducted between 7:00 and 14:00 during our bimonthly surveys. The number of observation hours per species ranged from 9 to 117 h, depending on the abundance of the plant species throughout the sampling months. During our observations, we recorded insect visitors, some of which may contribute to the pollination of certain plant species. However, adequately quantifying insect visitation was not feasible since insect sampling required smaller floral patches, shorter observation distances, and different collection methods. We noted the occurrence of these visitors to keep a record for future studies (Appendix S2).

### 2.5 Statistical Analysis

#### 2.5.1 Species Abundance

We used generalized linear mixed models (GLMM; Bolker et al. 2009) to analyze differences in the abundance of hummingbird and flowering plant species along the elevational gradient and between seasons. The number of individuals and flowers per sampling point were set as response variables. Fixed factors included site (low‐, mid‐, and high‐elevation), season (dry and rainy), and year; random factors included point counts and transects. GLMM were fitted with the negative binomial distribution for hummingbird, plant, and flower data to account for overdispersion. We used Tukey HSD for post hoc mean comparisons. These analyses were performed with the “glmer.nb” function from R package MASS (Ripley et al. 2016), “glht” function from R package multcomp (Hothorn et al. 2016), and “simulateResiduals” and “testOutliers” functions from R package DHARMa (Hartig and Hartig 2022; R Core Team 2023). We estimated the variance inflation factor (VIF) to test for collinearity between variables, where values of VIF < 10 indicate no collinearity, and values > 10 indicate multicollinearity (Dormann et al. 2013). None of the variables had collinearity problems (VIF < 5). We employed the “vifstep” function from R package usdm (Naimi et al. 2014; R Core Team 2023).

#### 2.5.2 Species Diversity

Alpha diversity was assessed through Hill's numbers (Chao, Chiu, and Jost 2014). The effective number of species was calculated for three diversity (^q^D) orders: ^0^D, ^1^D, and ^2^D, where the *q* parameter determines their sensitivity to relative abundance (Chao and Jost 2015). The order *q* = 0 (observed species richness) does not consider species abundances, whereas *q* = 1 (exponential Shannon entropy) weights taxa according to their frequency, and *q* = 2 (inverse of the Simpson index) indicates the effective number of dominant species, downplaying the impact of rare species (Chao and Jost 2012). Generalized linear models (GLM) were used to evaluate differences in Hill's numbers among sites and seasons. These analyses were performed using the “hill taxa” function from the R package hillR (Li 2018). Pairwise differences were evaluated with the Tukey HSD test. We obtained taxonomic beta diversity across the elevational gradient using the Sorensen (presence/absence) and Bray–Curtis (abundance) dissimilarity indexes. These were computed with the “beta.pair” function from the R package betapart (Baselga and Orme 2012). To visualize patterns of similarity across elevations and seasons, a non‐metric multidimensional scaling ordination (NMDS) was performed with the “metaMDS” function from the R package vegan (Oksanen 2013; R Core Team 2023).

#### 2.5.3 Interaction Networks

We constructed quantitative matrices using data from focal observations conducted at each elevation to evaluate the structure of plant–hummingbird interactions. We used visitation rates as a measure of interaction strengths. To obtain visitation rates for each plant species, we divided the number of flowers visited by each hummingbird species by the number of observation hours. We built interaction networks with the R package bipartite (Dormann, Gruber, and Fründ 2008), and quantified their main topological properties, including connectance, network specialization, nestedness, and modularity. Additionally, we identified core and periphery species.

Connectance (C) is the realized proportion of all possible interactions (Jordano 1987) and is defined as *C* = *I/(A·P)*, where *I* is the total number of interactions, *A* is the number of plant species, and *P* is the number of pollinator species; values closer to 1 indicate higher connectance. Network specialization ($H_{2}^{\prime }$) ranges from 0 for the most generalized (i.e., maximum niche overlap) to 1 for the most specialized network (i.e., no niche overlap; Blüthgen et al. 2007). We obtained the $H_{2}^{\prime }$ and the standardized specialization index (${∆H}_{2}^{\prime }$ = observed value—mean value of randomized networks; Schleuning et al. 2012); the latter accounts for differences in the size network. Nestedness quantifies the degree to which species in a network interact in a non‐random way. In a nested network, specialist species tend to interact with a subset of species that also interact with the more connected generalist species (Bascompte et al. 2003). We obtained the metric WNODF, which incorporates species abundance data (Almeida‐Neto and Ulrich 2011); this index ranges from 0 for non‐nested networks to 100 for perfect nestedness. We estimated connectance, WNODF, and specialization values with the “networklevel” function from the R package bipartite (Dormann, Gruber, and Fründ 2008). Modularity (*Q*) measures the extent to which groups within a network interact more frequently and intensely with each other than with other species outside their group (Olesen et al. 2007). This metric ranges from 0 (no modular structure) to 1, which is equivalent to the maximum degree of modularity (Dormann and Strauss 2014). We used Beckett's algorithm, which facilitates the detection of modules in weighted bipartite networks (Beckett 2016). Since the algorithm is stochastic, the arrangement of modules can vary across runs. Therefore, we retained the module structure with the highest *Q* value after 50 independent runs as the optimal (Maruyama et al. 2014); for this, we used the “computeModules” function (Dormann and Strauss 2014) from the R package bipartite (Dormann, Gruber, and Fründ 2008). The statistical significance of the metrics was analyzed by comparing the observed values to null models generated with Patefield's r2dtable algorithm (Patefield 1981), which is suitable for weighted networks; this keeps the sums of rows and columns constant in the random matrices, matching those in the observed network. We generated 1000 random matrices for connectance, WNODF, and specialization and 100 randomizations for modularity. We also obtained *Z*‐scores (observed‐mean [null]/SD [null]) of these metrics, and we evaluated the statistical significance using *z*‐tests.

We also employed combined nestedness, which considers the effect of connectance and network size on nestedness. This metric is known as NODF_*c*_ = NODF_*n*_/(*C*·log(*S*)), where NODF_*n*_ = NODF/max(NODF). In this formula, *C* represents connectance, *S* is the geometric mean of the number of species (log(*S*) allows NODF_*c*_ to be independent of network size), NODF is the raw NODF value for the network, and max(NODF) is the maximum nestedness of a network with the same number of species and links as the focal network, subject to the constraint that every species has at least one link (Song, Rohr, and Saavedra 2017). NODF_*c*_ replaces the *z*‐score. We used the algorithm quality = 2 for a better metric estimation (Song, Rohr, and Saavedra 2017). For these calculations, we used the “NODF_*c*_” function from R package maxnodf (Hoeppke and Simmons 2021).

The core–periphery metric measures the composition of core species a subgroup of densely connected species surrounded by peripherical species minorly connected (Miele, Ramos‐Jiliberto, and Vázquez 2020). We used the “CPness” function in the R package econetwork (Dray et al. 2020), for weighted networks. The core‐peripheriness (CPness) function is defined as CPness = (E_11_ + E_12_ + E_21_)/E, where E_*ij*_ is the number of interactions (edges) or the sum of weights for each block (E_*ij*_ for block *ij*) or the entire network (E) (Dray et al. 2020; Martín‐González et al. 2020; Miele, Ramos‐Jiliberto, and Vázquez 2020).

Beta diversity metrics for interactions were estimated with the Whittaker dissimilarity index. We calculated two additive components of beta diversity (*β*_WN_): species composition turnover (*β*_ST_) and interaction rewiring (*β*_OS_) across elevation and season networks. Additionally, we calculated the relative contribution of compositional differences given by (*β*_ST_/*β*_WN_). The dissimilarity index ranges between 0 and 1, where *β*_ST_/*β*_WN_ > 50% values indicate a high species turnover. Values of *β*_ST_/*β*_WN_ < 50% indicate a low species turnover and a higher influence of species rewiring. For these analyses, we used the “betalink” and “commondenom” functions implemented in the R package bipartite (Dormann, Gruber, and Fründ 2008).

#### 2.5.4 Influence of Ecological and Phylogenetic Factors on Interaction Frequencies

We built a phylogeny for the plant and hummingbird communities recorded at Nevado de Colima to evaluate the influence of phylogenetic relatedness on interaction frequencies. The plant phylogeny (*n* = 32) was built using the R package V.PhyloMaker (Jin and Qian 2019) based on Smith and Brown's (2018) dated phylogeny of seed plants. The “phylomaker” function automatically adjusts branch lengths to reflect the relative divergence times of input species. For the hummingbird community, we pruned the McGuire et al. (2014) phylogeny to include only the species recorded in our study, maintaining the original branch lengths. We employed phylogenetic GLMM (PGLMM) to examine the role of hummingbird and floral abundances, phenological overlap, and morphological matching (Euclidean distance between corolla and bill length/curvature) on interaction frequencies (pollinator visitation rates by each hummingbird species to each plant species). For morphological matching, two variables were included in the model, the length and curvature of flowers and bills. We added one‐third of the exposed bill culmen to correct tongue extension in hummingbird bill length estimates (Vizentin‐Bugoni, Maruyama, and Sazima 2014). We treated the strengths of pairwise interactions (visitation rates) as the dependent variable. We incorporated phylogenies into the models to account for anticipated covariances among these interactions and species traits. The models incorporated hierarchical and phylogenetic covariance matrices, which involved integrating phylogenetic relationships and traits to investigate whether closely related species displayed similar visitation patterns (phylogenetic attraction) or dissimilar patterns (phylogenetic repulsion). Random terms were integrated into the models to account for these relationships (Ives 2018). Visitation rates were log‐transformed to normalize residuals. Species traits were log‐transformed and Z‐transformed, with means centered at 0 and standard deviations set to 1, facilitating the interpretation of coefficients in the models as effect sizes. Separate PGLMM were constructed for the three elevations and seasons using the “pglmm” function from the R package phyr (Li et al. 2020). Models were evaluated with the Akaike criterion (AIC), and residuals were analyzed with the Kolmogorov–Smirnov test to assess normality. All analyses were performed with R software (R Core Team 2023).

## 3 Results

### 3.1 Richness, Diversity, and Abundance Patterns

**FIGURE 2:**
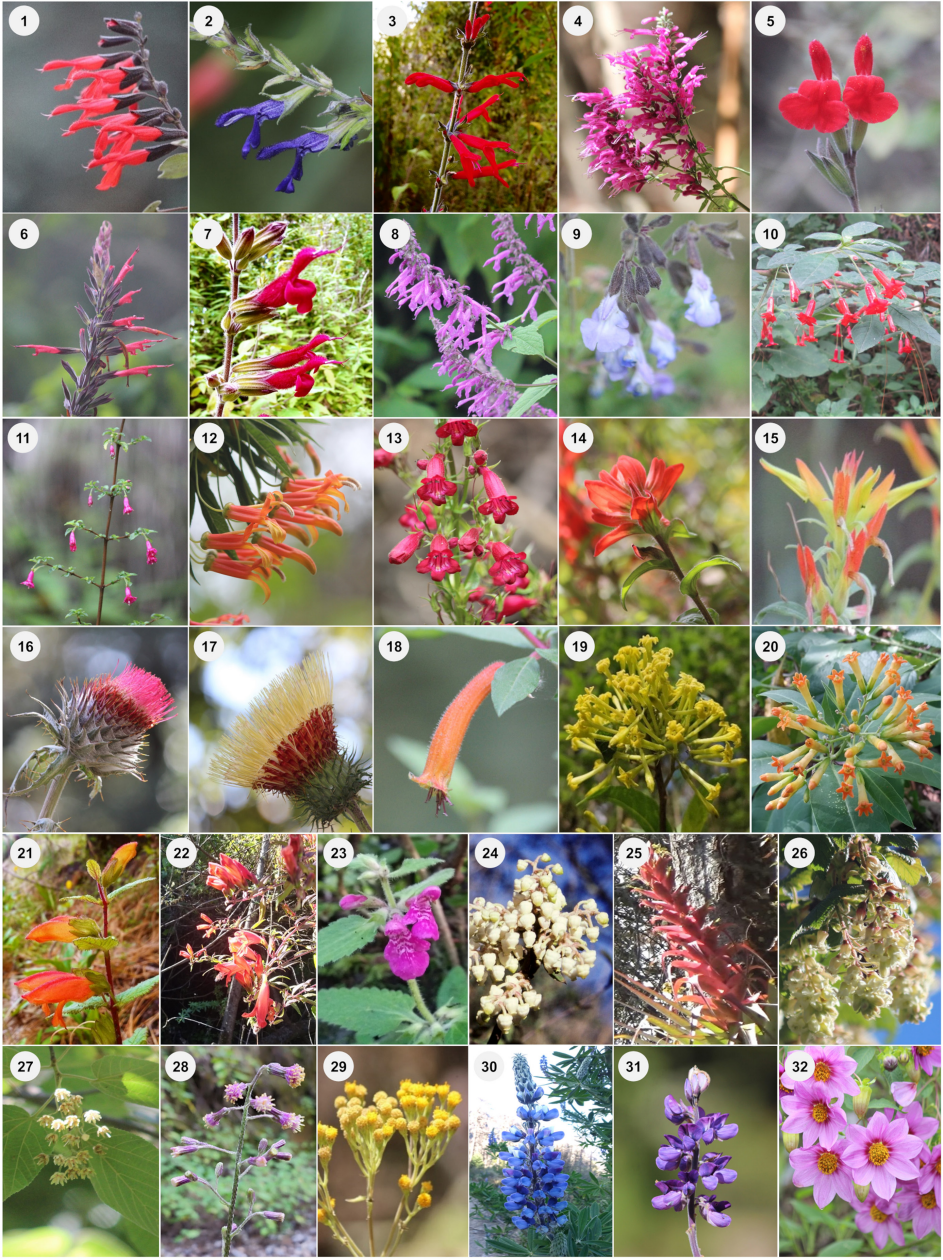
Plant species as floral resources for hummingbirds at Nevado de Colima Volcano National Park, México. (1) *Salvia gesneriiflora*, (2) *S. mexicana*, (3) *S. elegans*, (4) *S. iodantha*, (5) *S. microphylla*, (6) *S. longistyla*, (7) *Salvia* sp., (8) *S. purpurea*, (9) *S. ramamoorthyana*, (10) *Fuchsia cylindracea*, (11) *F. microphylla*, (12) *Lobelia laxiflora*, (13) *Penstemon roseus*, (14) *Castilleja cryptandra*, (15) *C. tenuiflora*, (16) *Cirsium ehrenbergii*, (17) *C. jaliscoense*, (18) *Cuphea watsoniana*, (19) *Cestrum laxum*, (20) *C. thyrsoideum*, (21) *Lamourouxia macrantha*, (22) *L. xalapensis*, (23) *Stachys pilosissima*, (24) *Arbutus xalapensis*, (25) *Tillandsia bourgaei*, (26) *Ribes ciliatum*, (27) *Tilia americana* var. *mexicana*, (28) *Senecio callosus*, (29) *Roldana angulifolia*, (30) *Lupinus reflexus*, (31) *L. montanus*, and (32) *Dahlia tenuicaulis*. Photographs by Eugenia Sentíes.

**FIGURE 3:**
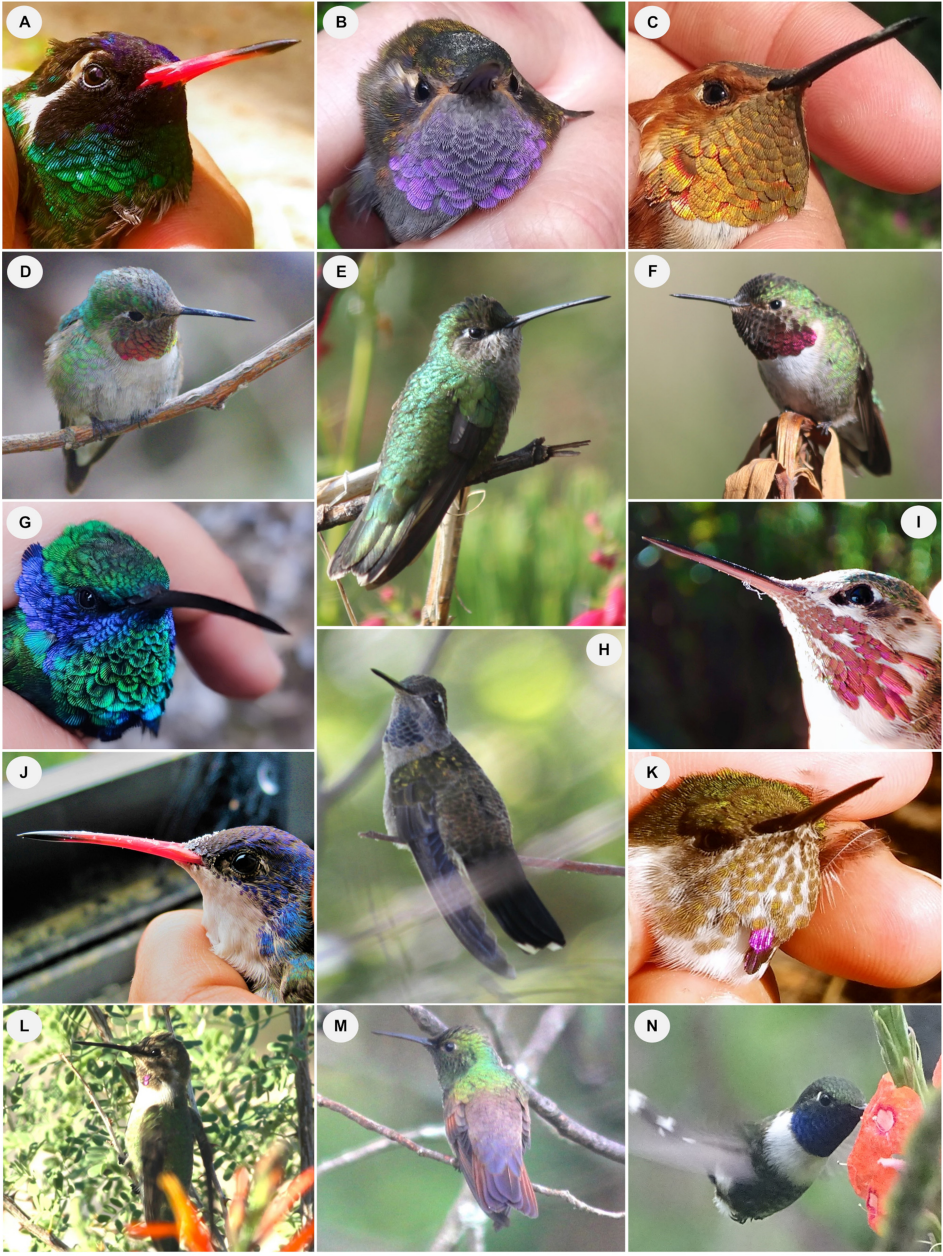
Hummingbird species recorded at Nevado de Colima Volcano National Park, México. (A) *Basilinna leucotis*, (B) *Lampornis amethystinus*, (C) *Selasphorus rufus*, (D) *Archilochus colubris*, (E) *Eugenes fulgens*, (F) *Selasphorus platycercus*, (G) *Colibri thalassinus*, (H) *Lampornis clemenciae*, (I) *Selasphorus calliope*, (J) *Ramosomyia violiceps*, (K) *Selasphorus heloisa*, (L) *Calypte costae*, (M) *Saucerottia beryllina*, and (N) *Tilmatura dupontii*. Photographs by Eugenia Sentíes, except photograph (N) by Sergio Díaz‐Infante.

We documented 32 plant species – belonging to 18 genera and 13 families – that were used as nectar resources by hummingbirds throughout the study period (Figure 2; Appendix S3). Most species (66%) had floral phenotypes that corresponded to the hummingbird pollination syndrome; the remaining species corresponded to insect pollination syndromes (*sensu* Faegri and van der Pijl 1979). The richness of blooming plants was 21, 27, and 8 species at low, mid, and high elevations along the gradient, respectively. These included some blooming species present at multiple elevations. We recorded 14 species of hummingbirds belonging to 10 genera within four monophyletic groups in the Trochilinae subfamily (Figure 3; Appendix S4). Three species are resident at the study site, while the rest are migratory: six elevational migrants and five latitudinal migrants. We recorded 12 hummingbird species in both the low‐ and mid‐elevation forests and nine species in the high‐elevation forest. Across elevations and forest types over the 2‐year period, the plant community interacting with hummingbirds included 30 species that bloomed during the dry season and 26 during the rainy season. The hummingbird community consisted of 13 species in the dry season and 11 in the rainy season. In the latter, one species, *S. calliope*, was recorded exclusively through mist‐netting; therefore, it was not included in the rainy‐season interaction network.

Alpha diversity for blooming plants species differed significantly among elevations (^0^D: $X_{2,6}^{2}$ = 19.65, *p* < 0.001; ^1^D: *F*_2,6_ = 18.94, *p* = 0.002; ^2^D: *F*_2,6_ = 17.93, *p* = 0.002) (Table 1), showing a significant decrease in the highest elevation forest (Appendix S5). For hummingbirds, the highest alpha diversity values were observed at the mid‐elevation forest, but there were no significant differences among elevations (^0^D: $X_{2,6}^{2}$ = 1.74, *p* = 0.41; ^1^D: *F*_2,6_ = 2.25, *p* = 0.18; ^2^D: *F*_2,6_ = 2.09, *p* = 0.2) (Table 1; Appendix S5). Richness and diversity differed between seasons, for both blooming plants (^0^D: $X_{1,10}^{2}$ = 9.75, *p* = 0.001; ^1^D: *F*_1,10_ = 20.64, *p* = 0.001; ^2^D: *F*_1,10_ = 22.56, *p* < 0.001), and hummingbirds (^0^D: $X_{1,10}^{2}$ = 4.73, *p* = 0.02; ^1^D: *F*_1,10_ = 14.70, *p* = 0.003; ^2^D: *F*_1,10_ = 7.94, *p* < 0.01). In all cases, diversity values were higher for the dry season (Table 1).

**TABLE 1:** Elevational and seasonal alpha diversity metrics for the interacting plant and hummingbird communities at Nevado de Colima Volcano National Park, México.

Group	Hill's numbers	Elevation			Season	
		Low	Mid	High	Dry	Rainy
Blooming plants	^0^ d	21 ± 1.20 ^a^	27 ± 1.20 ^a^	8 ± 0.88 ^b^	30 ± 1.38 ^a^	26 ± 1.31 ^b^
	^1^ d	7.3 ± 0.41 ^a^	5.8 ± 0.84 ^a^	2.1 ± 0.50 ^b^	9.5 ± 0.26 ^a^	4.5 ± 1.07 ^b^
	^2^ d	5.7 ± 0.41 ^a^	4.2 ± 0.64 ^a^	1.7 ± 0.32 ^b^	7.2 ± 0.17 ^a^	3.2 ± 0.82 ^b^
Hummingbirds	^0^ d	12 ± 1.15 ^a^	12 ± 0.66 ^a^	9 ± 0.88 ^a^	13 ± 1 ^a^	11 ± 0.60 ^b^
	^1^ d	2.7 ± 0.38 ^a^	4.4 ± 0.22 ^a^	3.7 ± 0.87 ^a^	4.4 ± 0.41 ^a^	2.5 ± 0.24 ^b^
	^2^ d	1.8 ± 0.22 ^a^	3.1 ± 0.39 ^a^	3 ± 0.75 ^a^	3.3 ± 0.35 ^a^	2.1 ± 0.27 ^b^

*Note:* Elevations are associated with three forest types: Low (pine‐oak; 2400–2600 m), mid (fir 2800–3100 m), high (subalpine; 3400–3700 m). Hill's numbers for alpha diversity: ^0^ d: Richness observed; ^1^ d: Shannon diversity (abundance); ^2^ d: Simpson's diversity (dominant species). Means with different superscripts (a/b) are statistically different between elevations and seasons (Tukey HSD test at *p* ≤ 0.05).

Beta diversity analyses for blooming plants revealed a high species turnover along the elevational gradient, with dissimilarity ranging from 20% to 96% based on presence/absence data, and from 75% to 96% when considering species abundance (Table 2); a distance‐decay pattern was observed from the low pine‐oak forest to the high‐elevation subalpine forest (Appendix S6). In contrast, for hummingbirds, species turnover across elevations was moderate, with dissimilarity values ranging from 16% to 23% for presence/absence data, and from 25% to 34% when considering species abundance (Table 2; Appendix S6). Seasonal turnover was low for both groups based on presence/absence data (14%–16%); however, when accounting for species abundances, turnover was notably higher for blooming plants (77%) than for hummingbirds (35%) (Table 2; Appendix S6).

**TABLE 2:** Elevational and seasonal beta diversity metrics for the interacting plant and hummingbird communities at Nevado de Colima Volcano National Park, México.

Group	Elevation				Season			
	Elev.1	Elev.2	Sorensen dissimilarity	Bray–Curtis dissimilarity	Seasons		Sorensen dissimilarity	Bray–Curtis dissimilarity
Blooming plants	Low	Mid	20%	75%	Dry	Rainy	14%	77%
	Low	High	57%	75%				
	Mid	High	75%	96%				
Hummingbirds	Low	Mid	16%	25%	Dry	Rainy	16%	35%
	Low	High	23%	32%				
	Mid	High	23%	34%				

*Note:* Elevations are associated with three forest types: Low (pine‐oak; 2400–2600 m), mid (fir 2800–3100 m), high (subalpine; 3400–3700 m). Sorensen (presence/absence) and Bray–Curtis (abundance) dissimilarity indexes values (0–1) are shown in percentage.

**FIGURE 4:**
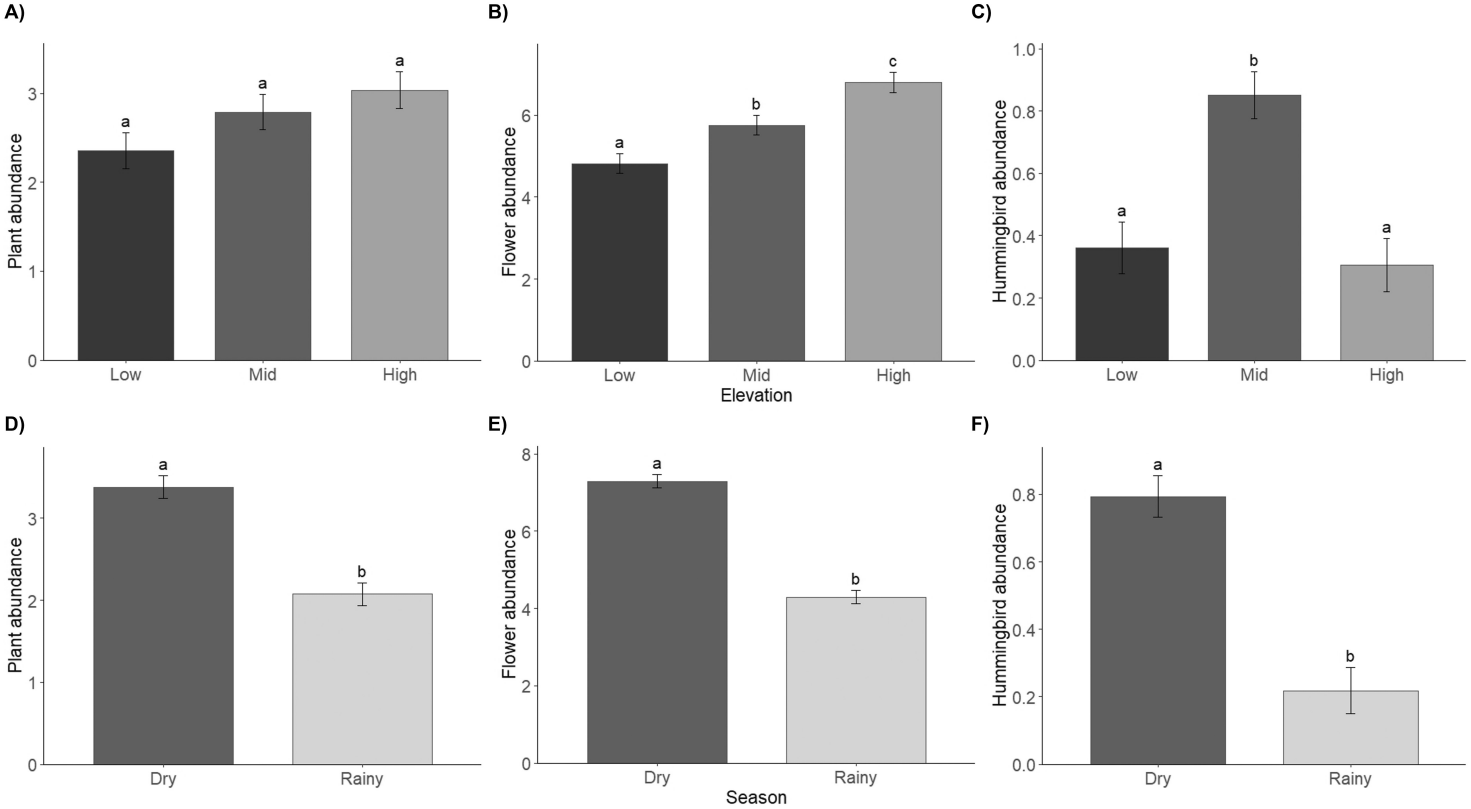
Elevational and seasonal species richness and abundance at Nevado de Colima Volcano National Park, México. For Blooming plants (A, D); open flowers (B, E); and hummingbirds (C, F). Error bars represent standard errors, and letters on error bars indicate statistical differences after Tukey pairwise comparisons (*p* < 0.05).

The abundance of blooming individuals did not change with elevation (*X* ^2^ = 5.5, df = 2, *p* = 0.06) (Figure 4A), but flower abundance did (*X* ^2^ = 34.3, df = 2, *p* < 0.001), being significantly greater in the high‐elevation forest (Figure 4B). Blooming plant and flower abundance differed between seasons (*X* ^2^ = 71.8, df = 1, *p* < 0.001; *X* ^2^ = 259.3, df = 1, *p* < 0.001, respectively), with greater abundances during the dry season (Figure 4D,E). Hummingbird abundance changed across elevations (*X* ^2^ = 30.5, df = 2, *p* < 0.001), being significantly higher in the mid‐elevation forest (Figure 4C). Hummingbird abundance differed between seasons (*X* ^2^ = 44.4, df = 1, *p* < 0.001) and was greater during the dry season (Figure 4F). Species abundance varied between years for both blooming plants (*X* ^2^ = 11.1, df = 1, *p* = 0.0008) and hummingbirds (*X* ^2^ = 6.7, df = 1, *p* = 0.009), with consistently higher abundance in the first sampling year. However, flower abundance did not significantly differ between years (*X* ^2^ = 3.1, df = 1, *p* = 0.08).

### 3.2 Species Phenology

Phenological sampling over 2 years revealed a greater proportion of flowering species and individuals during the dry season (November, January, and March) compared to the rainy season (May, July, and September) (Appendix S7). Some plant species bloomed year‐round at varying intensities, while others were restricted to specific months (Appendix S7). Hummingbird species abundances, independently determined by point counts, also varied through the year, but three species were present across both sampling years (*Basilinna leucotis*, *Eugenes fulgens*, and *Lampornis amethystinus*). The remaining species were either elevational or latitudinal migrants (Appendix S7).

### 3.3 Plant–Hummingbird Interaction Networks

**FIGURE 5:**
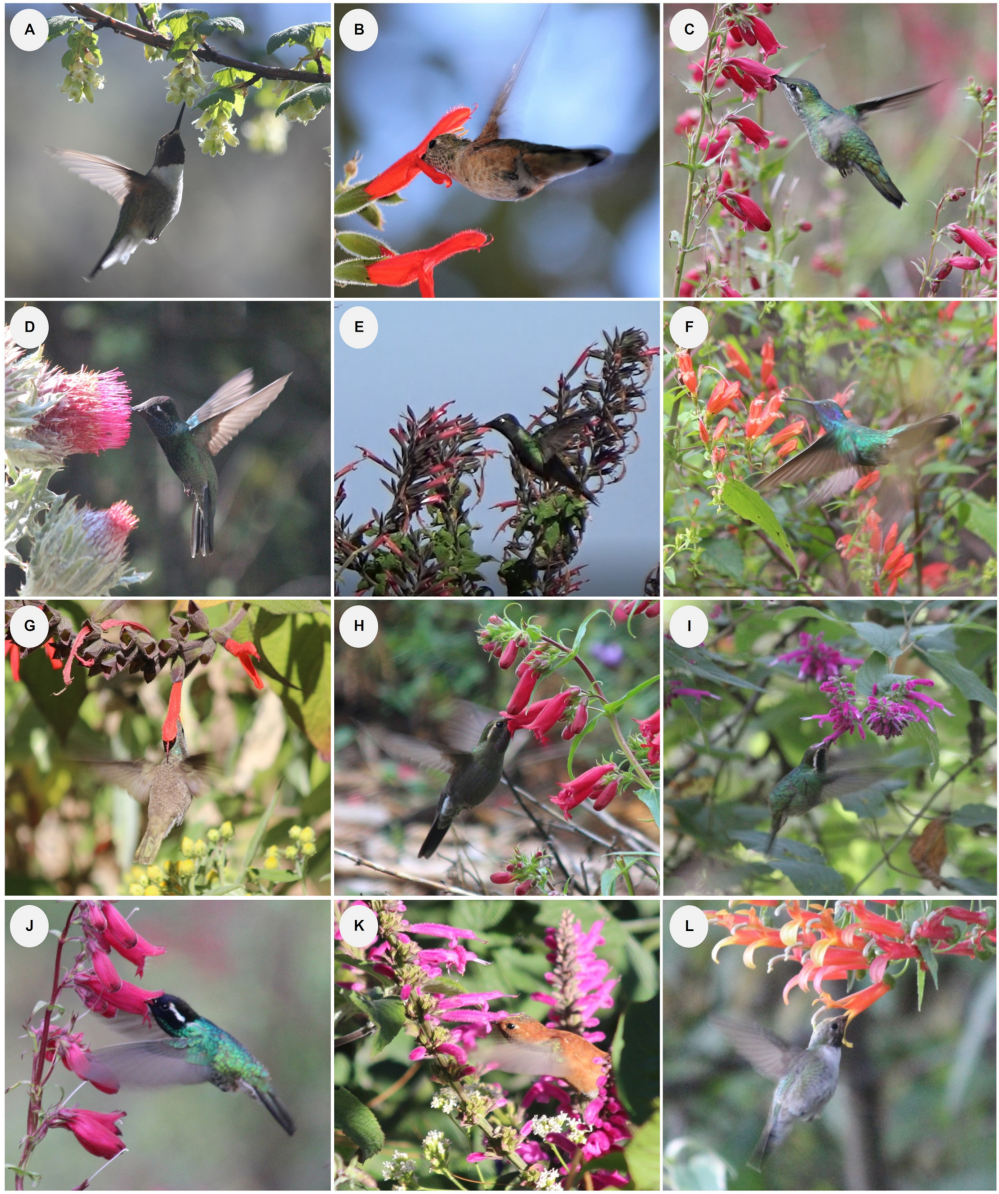
Examples of plant–hummingbird interactions recorded at Nevado de Colima Volcano National Park, México. (A) *S. platycercus* ♂—*R. ciliatum*, (B) *S. platycercus* ♂—*S. gesneriiflora*, (C) *E. fulgens* ♀—*P. roseus*, (D) *E. fulgens* ♂—*C. ehrenbergii*, (E) *E. fulgens* ♂—*S. longistyla*, (F) *C. thalassinus*—*L. xalapensis*, (G) *R. violiceps*—*S. gesneriiflora*, (H) *L. amethystinus* ♂—*P. roseus*, (I) *B. leucotis* ♀—*S. iodantha*, (J) *B. leucotis* ♂—*P. roseus*, (K) *S. rufus* ♂—*S. iodantha*, and (L) *A. colubris* ♂—*L. laxiflora*. See the full species scientific name in Figures 2 and 3. Photographs by Eugenia Sentíes.

We recorded 125 unique plant–hummingbird interactions across the elevational gradient (Figure 5), with network structures varying across space and time (Table 3). The number of blooming plant and hummingbird species presented in interaction networks may be lower than the total species richness reported by elevation or season. The latter considered blooming species that served as nectar sources for hummingbirds in at least one forest type or season, regardless of whether they interacted with hummingbirds at the specific site or season they were recorded. The number of blooming plant and hummingbird species in the networks corresponds to interactions specifically recorded at each elevation and season, where interactions with hummingbirds may have been recorded only at one elevation (Table 3).

**TABLE 3:** Plant–hummingbird interaction network metrics across three elevations and two seasons at Nevado de Colima Volcano National Park, México.

Network metrics	Elevation			Season	
	Low	Mid	High	Dry	Rainy
Blooming plant species	16	22	6	24	23
Hummingbird species	10	11	9	13	10
Size	160	242	54	312	230
Links per species	2.15	2.64	1.07	2.65	1.58
Connectance (C)	0.35* (−6.7)	0.36* (−8.7)	0.30* (−8.5)	0.31* (−6.8)	0.23* (−12.9)
Nestedness (WNODF)	39.51* (−4.8)	45.10* (−6.8)	35.62* (−5.5)	54.65* (−3.2)	25.95* (−7.5)
Nestedness (nodf_*c*_ )	1.97	1.91	3.33	2.23	2.94
Specialization ( $H_{2}^{\prime }$ )	0.38* (37.8)	0.39* (77.6)	0.65* (71.9)	0.29* (47.3)	0.59* (59.1)
Specialization ( $∆H_{2}^{\prime }$ )	0.33	0.35	0.63	0.25	0.54
Modularity (*q*)	0.40* (27.0)	0.38* (42.6)	0.45* (25.9)	0.35* (38.1)	0.47* (38.4)
Modularity (Δ*q*)	0.31	0.31	0.39	0.27	0.37

*Note:* Elevations are associated with three forest types: Low (pine‐oak; 2400–2600 m), mid (fir 2800–3100 m), high (subalpine; 3400–3700 m). In parentheses, positive and negative *Z*‐scores indicate that the empirical network metric was higher or lower, respectively, than the mean metric obtained from the null model. Asterisks show significant deviation from the null model (*p* < 0.05). Δ standardized metrics resulted from observed value – mean values of randomized networks.

**FIGURE 6:**
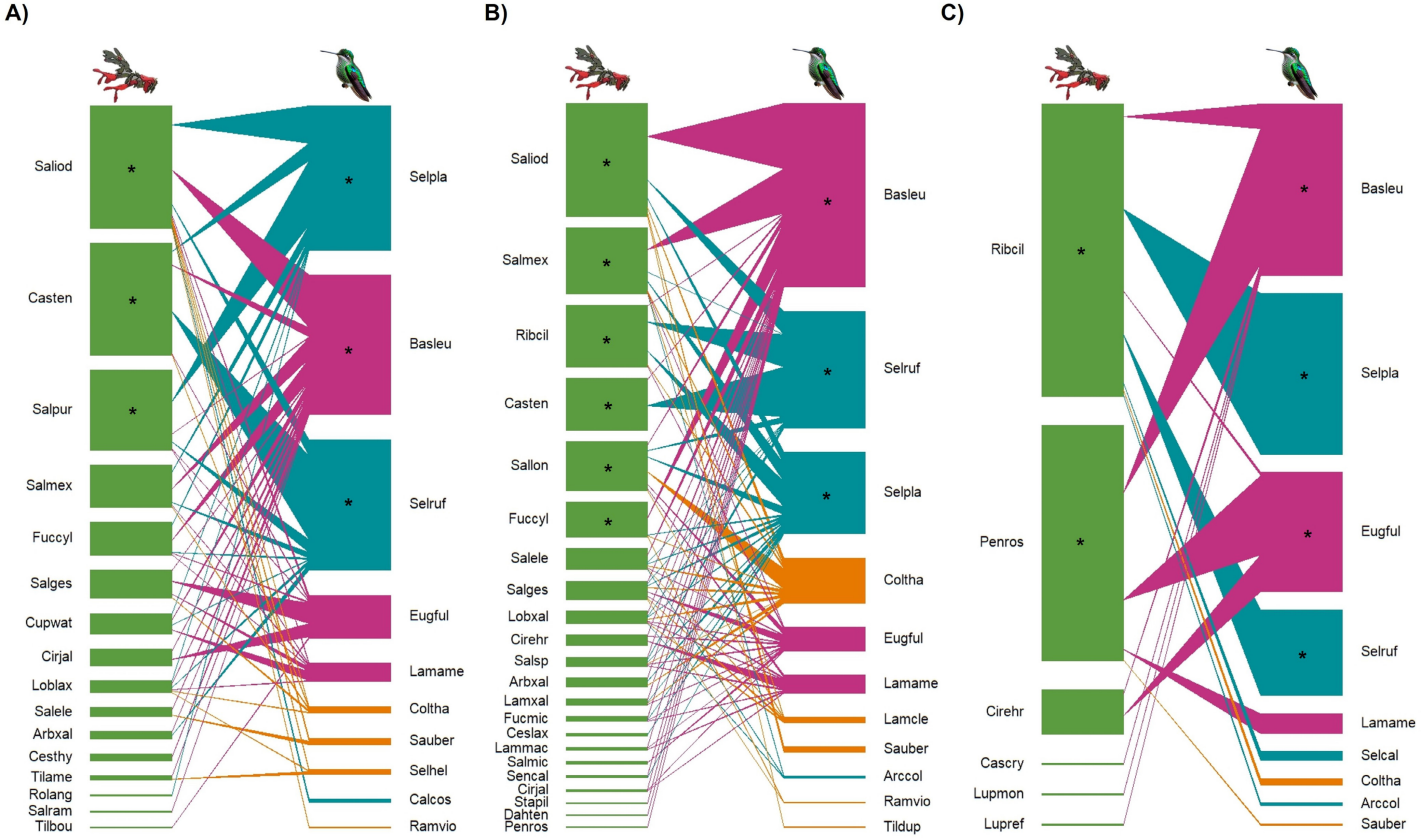
Plant–hummingbird interaction networks along a high mountain elevational gradient at Nevado de Colima Volcano National Park, México. (A) Low site, pine‐oak forest. (B) Mid site, fir forest. (C) High site, subalpine forest. Green bars represent the plants. For hummingbirds, different colors represent their seasonality: Residents (purple), latitudinal migrants (aquamarine), and elevational migrants (orange). Core species are represented by asterisks. Plant species codes: *Arbutus xalapensis* (Arbxal), *Castilleja cryptandra* (Cascry), *C. tenuiflora* (Casten), *Cestrum laxum* (Ceslax), *C. thyrsoideum* (Cesthy), *Cirsium ehrenbergii* (Cirehr), *C. jaliscoense* (Cirjal), *Cuphea watsoniana* (Cupwat), *Dahlia tenuicaulis* (Dahten), *Fuchsia cylindracea* (Fuccyl), *F. microphylla* (Fucmic), *Lamourouxia macrantha* (Lammac), *L. xalapensis* (Lamxal), *Lobelia laxiflora* (Loblax), *Lupinus reflexus* (Lupref), *L. montanus* (Lupmon), *Penstemum roseus* (Penros), *Ribes ciliatum* (Ribcil), *Roldana angulifolia* (Rolang), *Salvia elegans* (Salele), *S. gesneriiflora* (Salges), *S. iodantha* (Saliod), *S. longistyla* (Sallon), *S. mexicana* (Salmex), *S. microphylla* (Salmic), *S. purpurea* (Salpur), *S. ramamoorthyana* (Salram), *Salvia* sp. (Salsp), *Senecio callosus* (Sencal), *Stachys pilosissima* (Stapil), *Tillandsia bourgaei* (Tilbou), and *Tilia americana* var. mexicana (Tilame). Hummingbird species codes: *Archilochus colubris* (Arccol), *Basilinna leucotis* (Basleu), *Calypte costae* (Calcos), *Colibri thalassinus* (Coltha), *Eugenes fulgens* (Eugful), *Lampornis amethystinus* (Lamame), *L. clemenciae* (Lamcle), *Ramosomyia violiceps* (Ramvio), *Saucerottia beryllina* (Sauber), *Selasphorus calliope* (Selcal), *S. heloisa* (Selhel), *S. platycercus* (Selpla), *S. rufus* (Selruf), *and Tilmatura dupontii* (Tildup). Illustrations: *Salvia* by Eugenia Sentíes; hummingbird image was generated by ChatGPT (Open AI 2024).

**FIGURE 7:**
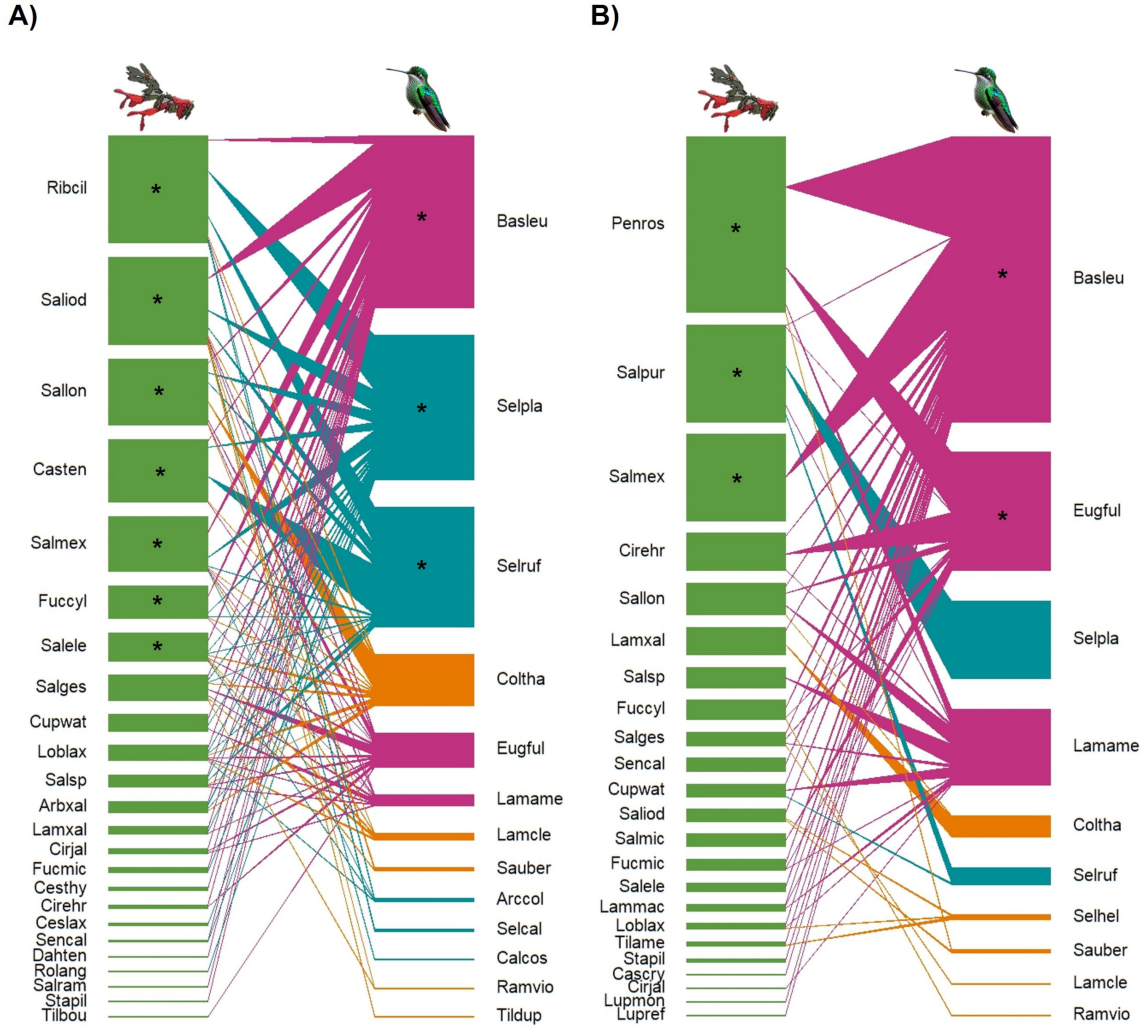
Plant–hummingbird interaction networks recorded in the dry and rainy seasons at Nevado de Colima Volcano National Park, México. (A) Dry, (B) rainy. Green bars represent the plants. For hummingbirds, different colors represent their seasonality: Residents (purple), latitudinal migrants (aquamarine), and elevational migrants (orange). Core species are represented by asterisks. See plant and hummingbird species codes in Figure 6. Illustrations: *Salvia* by Eugenia Sentíes; hummingbird image was generated by ChatGPT (Open AI 2024).

Networks were largest at the mid‐elevation fir forest (Table 3; Figure 6) and during the dry season (Table 3; Figure 7). All networks were significantly nested with the WNODF metric, and standardized nestedness (NODF_*c*_) values were similar across elevations and seasons (Table 3). All networks had significant connectance and modularity values that were similar across elevations and seasons. However, network specialization was higher in the high‐elevation subalpine forest and during the rainy season, than at other elevations and seasons (Table 3). Core plant species varied across elevations and seasons (Figures 6 and 7). *B. leucotis* was a core hummingbird species across elevations and seasons (Figures 6 and 7), *Selasphorus platycercus* and *S. rufus* were core species across elevations and during the dry season (Figures 6 and 7A), and *E. fulgens* was core at the high‐elevation forest and during the rainy season (Figures 6C and 7B).

The beta diversity metric for interaction networks showed a high interaction turnover (*β*_ST_/*β*_WN_: 75%–100%) across the elevational gradient. This indicates that plant–hummingbird interactions change as species composition shifts along the gradient (Table 4). In contrast, seasonal differences in plant–hummingbird interactions were better explained by the rewiring of interactions (*β*_ST_/*β*_WN_ = 47%), meaning that while species composition remains relatively stable, the interactions among species shift over time.

**TABLE 4:** Beta diversity metrics for plant–hummingbird interaction networks (*β*_WN_) at Nevado de Colima Volcano National Park, México.

Network 1	Network 2	*β*_st_	*β*_os_	*β*_wn_	*β*_st_ / *β*_wn_
Elevation					
Low	Mid	0.53	0.17	0.71	75.2
Low	High	1	0	1	100
Mid	High	0.80	0.05	0.84	94.3
Season					
Dry	Rainy	0.31	0.35	0.67	47

*Note:* Elevations are associated with three forest types: Low (pine‐oak; 2400–2600 m), mid (fir 2800–3100 m), high (subalpine; 3400–3700 m). Dissimilarity in species turnover (*β*_ST_) and shared species interactions (*β*_OS_). *β*_ST_/*β*_WN_ values express the relative contribution of these components to beta diversity (%). The dissimilarity index ranges from 0 to 1. Values of *β*_ST_/*β*_WN_ > 50% indicate high turnover, and values of *β*_ST_/*β*_WN_ < 50% indicate species rewiring.

### 3.4 Influence of Ecological and Phylogenetic Factors on Interactions Frequencies

We found a low but significant effect of flower abundance on the log‐transformed interaction frequencies at the low‐ and mid‐elevation forests. Hummingbird abundance did not influence elevational variation in interaction frequencies but had a small significant effect on visitation frequencies across seasons (Table 5). Phenological matching positively influenced interaction frequencies across elevations and seasons (Table 5). Morphological matching (measured through the variables of length and curvature of flowers and bills) significantly affected plant–hummingbird interaction frequencies at the low elevation and in the dry season. In this case, the statistics showing a negative association between flower and hummingbird traits indicate a greater correspondence between matched morphologies and visitation frequencies (Table 5).

**TABLE 5:** Phylogenetic generalized linear mixed model (PGLMM) results showing the effects of ecological and phylogenetic factors on plant–hummingbird interaction frequencies at Nevado de Colima Volcano National Park, México.

Effects	Elevation gradient						Season			
	Low		Mid		High		Dry		Rainy	
Fixed	ES	*Z*	ES	*Z*	ES	*Z*	ES	*Z*	ES	*Z*
Flower abundance	0.00007*	**2.1**	0.00003**	**2.8**	0.000	1	0.000	1.8	0.0000	1.4
Hummingbird abundance	0.000	0.009	0.004	1.5	0.006	0.8	0.003*	**2.2**	0.006*	**2.3**
Phenological overlap	0.4***	**4.3**	0.2***	**3.7**	0.3*	**2.1**	0.5***	**6.2**	0.3**	**3.3**
Morphological matching	−0.07*	**−2.4**	−0.03	−1.9	−0.01	−0.2	−0.05**	**−2.8**	−0.05	−1.8

Random	ES	LR	ES	LR	ES	LR	ES	LR	ES	LR
$\sigma_{a}^{2}$	0.2*	**1.8**	0.3***	**6.1**	0.000	−0.000	0.05	0.5	0.3**	**2.7**
$\sigma_{b}^{2}$	0.000	−0.000	0.000	−0.000	0.000	−0.000	0.000	−0.000	0.000	−0.000
$\sigma_{c}^{2}$	0.000	−0.000	0.000	−0.000	0.000	−0.000	0.000	−0.000	0.2*	**2**
$\sigma_{d}^{2}$	0.8***	**17**	0.4***	**9.9**	4***	**5.2**	0.9***	**18.8**	0.4*	**1.4**
$\sigma_{f}^{2}$	0.000	−0.000	0.000	−0.000	0.000	−0.000	0.000	−0.000	0.01	0.02
$\sigma_{g}^{2}$	0.000	−0.000	0.01	0.05	−0.000	−0.000	0.000	−0.000	0.000	−0.000
$\sigma_{h}^{2}$	0.000	−0.000	0.000	−0.000	0.3**	**3.3**	0.02	0.4	0.000	−0.000

*Note:* Elevations are associated with three forest types: Low (pine‐oak; 2400–2600 m), mid (fir 2800–3100 m), high (subalpine; 3400–3700 m). Estimated size value (ES) for fixed and random effects; *Z*‐scores for random effects; LR, likelihood ratio for random effects. ($\sigma_{a}^{2}$) Non‐phylogenetic, random variation in the frequencies of interactions established by hummingbirds; ($\sigma_{b}^{2}$) Variation in interaction frequencies explained by pollinator phylogenies, i.e., closely related hummingbird species share similar visitation rates. ($\sigma_{c}^{2}$) Variation in partner identity explained by pollinator phylogenies, i.e., closely related hummingbird species use the same plant species. ($\sigma_{d}^{2}$) Non‐phylogenetic, random variation in the frequency of interactions established by plants. ($\sigma_{f}^{2}$) Variation in interaction frequencies explained by plant phylogeny, i.e., closely related plant species have similar visitation rates; ($\sigma_{g}^{2}$) variation in partner identity explained by plant phylogenies, i.e., closely related plant species attract the same pollinator species; ($\sigma_{h}^{2}$) co‐phylogenetic component of variation, i.e., closely related hummingbird species are more likely to visit closely related plant species. Bold values show statistically significant effects at *p* < 0.05. Significance levels are indicated by asterisks (***≤ 0.0001, **≤ 0.001, *≤ 0.05).

The analysis of phylogenetic random effects indicated small and mostly non‐significant effects of phylogenetic relatedness in interaction frequencies or partner identity (Table 5). For plants, the random component of variation ($\sigma_{d}^{2}$) was significant across elevations and seasons, whereas the phylogenetic components of variation were negligible ($\sigma_{e}^{2}$, $\sigma_{f}^{2}$; Table 5). This indicates that closely related plant species do not share similar visitation frequencies, or attract the same species of hummingbirds more than would be expected by chance. For hummingbirds, non‐phylogenetic factors ($\sigma_{a}^{2}$) significantly influenced plant–hummingbird interaction patterns across low‐ and mid‐elevation forests, and for the dry‐season network (Table 5). In line with this, phylogenetic effects on hummingbird interaction frequencies were negligible across elevations and seasons ($\sigma_{b}^{2}$), indicating that closely related hummingbird species do not exhibit similar visitation frequencies. For the rainy‐season network, there was a significant phylogenetic effect on partner identity ($\sigma_{c}^{2}$), meaning that during this season, closely related species of hummingbirds tend to use the same plant species. Only for the high‐elevation network, there was a small but significant co‐phylogenetic effect ($\sigma_{h}^{2}$), meaning that closely related hummingbird species are more likely to visit closely related plant species in this environment (Table 5).

## 4 Discussion

### 4.1 Variation in Diversity and Community Composition

#### 4.1.1 Elevational Patterns

In this study, the diversity metrics of blooming plants used by hummingbirds were comparable in the pine‐oak and mid‐elevation fir forests, while a sharp decline was observed in the highest‐elevation subalpine forest. For hummingbirds, diversity metrics did not differ among elevations, but richness also started to decline in the subalpine forest. An assessment of previous studies along the mountain gradient associated with Nevado de Colima shows reduced elevational variation for plant and hummingbird richness. Specifically, the richness of blooming plant species used by hummingbirds remains strikingly similar (21–27 spp.) across most of the gradient, from the nearest protected lowland dry forest of Chamela (100 m) to the fir forest (up to 3200 m) (Díaz‐Infante, Lara, and Arizmendi 2020; Des Granges 1979; this study), dropping to eight species in the subalpine forest (this study). In contrast, seven species of hummingbirds have been reported in the lowland dry forests (Arizmendi and Ornelas 1990; Díaz‐Infante, Lara, and Arizmendi 2020), increasing to 12 species in low‐mid‐elevation dry habitats (Des Granges 1979) and remaining stable throughout the pine‐oak and fir forests (Des Granges 1979; this study), before starting to decline in the highest elevation subalpine forest (nine species). Our findings are consistent with previous research showing that diversity tends to decline above a certain elevation threshold in high mountains, likely due to the shift from humid habitats to drier or more extreme environments (e.g., Nogués‐Bravo et al. 2008; McCain and Grytnes 2010).

High beta diversity along mountain gradients has been documented in tropical and temperate regions for both plants (Dzekashu et al. 2022; Cordeiro et al. 2023; Minachilis et al. 2023; Martínez‐Roldán, Pérez‐Crespo, and Lara 2024) and hummingbirds (Weinstein et al. 2014; Maglianesi et al. 2015; Sonne et al. 2019; Guevara et al. 2023). Our findings support this pattern for plants only. Beta diversity values were relatively low for hummingbirds, with most species recorded at two or three elevations along the gradient. This may be attributed to the high mobility of hummingbirds, which fly up and down the mountain slope in response to changes in the phenology of their floral resources. In contrast, beta diversity metrics were high for plants along the elevational gradient. In tropical mountains, where species are adapted to specific thermal conditions and experience less pronounced seasonal variation compared to temperate regions, high species turnover along elevational gradients is expected (Janzen 1967; Ruggiero and Hawkins 2008; Fjeldså, Bowie, and Rahbek 2012), particularly for sessile organisms.

#### 4.1.2 Seasonal Patterns

Seasonal variation in diversity patterns associated with plant–pollinator interactions has been poorly documented in tropical mountain gradients. Our study found that the richness and abundance of blooming plants and hummingbirds were higher in the dry season across the three elevations. This result is consistent with records for mountain sites in western Mexico (Arizmendi 2001; Partida‐Lara et al. 2018) and South America (Weinstein and Graham 2017). Although temperatures are lower during the dry season, flowering is synchronized with the timing of arrival of resident and migratory hummingbirds at Nevado de Colima. Elevational migrants follow the shifting availability of floral resources (Levey and Stiles 1992; López‐Segoviano et al. 2018; Williamson and Witt 2021), while latitudinal migrants use this protected mountain refuge as a wintering and foraging site before returning to their northern breeding grounds. These results underscore the importance of high mountain forests for migratory hummingbird species and suggest that these birds may play a key role in the reproduction of highland plants.

### 4.2 Plant–Hummingbird Interaction Networks

#### 4.2.1 Elevational and Seasonal Patterns

The structure of interaction networks varied across the elevational gradient. All networks were significantly nested with the WNODF metric, and standardized nestedness metric (NODF_*c*_) was comparable across elevations and seasons. Connectance—the proportion of realized to potential interactions—was similar along the gradient, as reported for plant–hummingbird networks from other mountain sites (Sonne et al. 2020). In contrast, we found the highest specialization values in the subalpine forest, corresponding to the least diverse network. This pattern has also been reported for high‐elevation Andean plant–hummingbird networks (Watts et al. 2016; Pelayo et al. 2019, 2021; Sonne et al. 2019) but is not common in other plant–pollinator networks (e.g., Lara‐Romero et al. 2019; Classen et al. 2020). In this study, higher specialization in the subalpine forest may be driven by the abundant blooming of two plant species, *Ribes ciliatum* and *Penstemon roseus*, which serve as key resources for various hummingbird species. Additionally, *B. leucotis* is the exclusive hummingbird visitor of three plant species, *Castilleja cryptandra* and two primarily bee‐pollinated *Lupinus* species. Thus, specialization at this site is asymmetric, influenced by a small number of abundant plant and hummingbird species.

The high beta diversity of interactions along the elevational gradient was explained by a high interaction turnover, corresponding mostly to changes in plant species composition. This pattern has also been observed in plant–hummingbird networks from Andean mountain gradients (Graham and Weinstein 2018; Guevara et al. 2023) and other pollination networks (Carstensen et al. 2014; Lara‐Romero et al. 2019; Luna et al. 2023). Changes in temperature and other environmental factors contribute to narrow elevational distributions, species turnover, and distinct networks at different elevations, extending Janzen's (1967) hypothesis to ecological networks.

Changes in plant and pollinator phenology also influenced the structure of interaction networks (Chávez‐González et al. 2020). In our study, nestedness and connectance were similar between seasons, but network specialization was higher in the rainy season, a period of low blooming‐plant abundance at Nevado de Colima. The pattern of temporal specialization in plant–hummingbird interactions associated with low floral resource availability regardless of season has also been found in other studies (Tinoco et al. 2017; Souza et al. 2018). For example, in the Andean highlands, interactions established by long‐billed hummingbirds were more specialized during the rainy season (Tinoco et al. 2017). In contrast, in the Brazililian Pantanal and Cerrado habitats, plant–hummingbird interactions were more specialized during the dry season when floral resources were less abundant (Souza et al. 2018). Specialization in response to low flower availability may serve as a strategy to optimize resource use efficiency and reduce interspecific competition (Tinoco et al. 2017; Sargent, Groom, and Rico‐Guevara 2021). At Nevado de Colima, increased specialization was observed in the resident hummingbird *Colibri thalassinus*, and the migrants *S. platycercus* and *S. rufus* during the rainy season.

#### 4.2.2 Spatiotemporal Patterns

Interaction patterns across the elevational gradient were strongly determined by seasonal hummingbird movements and spatiotemporal fluctuations in floral resources. Species rewiring (i.e., shifting partner species), a common seasonal trend in pollination networks (Graham and Weinstein 2018; CaraDonna et al. 2017; Hervías‐Parejo et al. 2023; Cortés‐Flores et al. 2023), explained seasonal differences in plant–hummingbird interactions. The dry season was the period of greatest floral diversity and abundance, particularly in the mid‐ and high‐elevation forests, corresponding to the time resident hummingbirds overlapped with latitudinal and elevational migrants. During the rainy season, hummingbird and floral resources abundance declined in the low‐elevation (pine‐oak) and mid‐elevation (fir) forests, driving the movement of resident and some elevational migrant hummingbird species to the subalpine site, where the shrub *P. roseus* was abundant.

### 4.3 Core Species

The seasonal differences observed in species richness and abundance influence the importance of individual species in pollination networks, defining core species that connect and increase network robustness (Jordano, Bascompte, and Olesen 2003; Emer et al. 2016). We found that core plant species changed across elevations and seasons. *Fuchsia cylindracea*, *Castilleja tenuiflora*, and *Salvia* species (*S. iodantha*, *S. mexicana*, *S. longistyla*, and *S. purpurea*) were key species in pine‐oak and fir forests, providing important resources for hummingbirds. The genera *Salvia*, *Fuchsia*, and *Castilleja* have also been documented as important nectar sources for hummingbirds in other tropical and temperate mountain habitats (e.g., Wolf, Stiles, and Hainsworth 1976; Izquierdo et al. 2023). During the dry season, core plant species were the same as those mentioned for the elevation gradient (except for *S. purpurea* and adding to *S. elegans*). In the mid‐elevation fir and subalpine forest, the core species *R. ciliatum* is a significant nectar source for latitudinal migratory species, such as the threatened *S. rufus*. Other *Ribes* species have been recorded as valuable floral resources for hummingbirds in temperate zones (Magrach et al. 2020). During the rainy season, core plant species include two *Salvia* species (*S. purpurea*, *S. mexicana*), which are core species in the low‐ and mid‐elevation forests, respectively, and the endemic *P. roseus*, which is also a core species in the high‐elevation subalpine forest. The genus *Penstemon* also provides abundant floral resources for hummingbirds in other tropical and temperate highlands (Castellanos, Wilson, and Thomson 2003; Salas‐Arcos et al. 2019; Cardona, Lara, and Ornelas 2020). The four core hummingbird species in our study (*B. leucotis*, *E. fulgens*, *S. platycercus*, and *S. rufus*) are abundant and commonly found throughout Mexican mountain forests (Arizmendi 2001; Rodríguez‐Flores and Arizmendi Arriaga 2016; Martínez‐Roldán, Pérez‐Crespo, and Lara 2024). In our study, the widely distributed *B. leucotis* remained a core species across elevations and seasons. Widespread abundant species tend to have a generalist behavior owing to the greater possibilities of interacting with potential resources (Rodríguez‐Flores et al. 2019; Simmons et al. 2019). This condition also applies to the latitudinal migrants *S. platycercus* and *S. rufus*, core species across elevations and in the dry season. In contrast, the resident *E. fulgens* was a core species only in the subalpine forest and during the rainy season, primarily due to its frequent interaction with *P. roseus*.

### 4.4 Influence of Ecological and Phylogenetic Factors on Interactions Frequencies

#### 4.4.1 Ecological Factors

Neutral hypotheses assume that abundant species have more interaction partners and higher interaction frequencies (Vázquez et al. 2007; Krishna et al. 2008). In our study, flower abundance had a low but significant influence on the frequency of interactions at low‐ and mid‐elevation, where floral abundance varies among species. Hummingbird abundance also had a low significant effect on interaction frequencies for dry‐ and rainy‐season networks, which can be attributed to the seasonality of hummingbird species. Other studies in the tropics have found a moderate effect of abundance on plant–hummingbird interactions (Vizentin‐Bugoni, Maruyama, and Sazima 2014; Gonzalez and Loiselle 2016), compared to the influence of temporal overlap and morphological matching. Phenology is a major driver of interaction frequencies, determined by the temporal coincidence of interaction partners (Chávez‐González et al. 2020; Sonne et al. 2020). Accordingly, we found that phenology played a key role in interaction frequencies both across the elevational gradient and between seasons, as has been documented for plant–hummingbird interactions from other tropical mountains (Vizentin‐Bugoni, Maruyama, and Sazima 2014; Gonzalez and Loiselle 2016; Martín‐González et al. 2018; Chávez‐González et al. 2020).

Morphological coupling has been proposed as an important driver of plant–pollinator interactions, as it promotes pollination efficiency and enhances the hummingbirds' ability to extract nectar (Temeles et al. 2002; Maglianesi et al. 2014). However, we found that the effect of morphological matching was significant only for low‐elevation pine‐oak forest and dry season networks. In this forest, floral diversity was higher, and hummingbird species with varying bill sizes, particularly medium and small, interacted with multiple plant species (e.g., *Salvia*, *Cestrum*, and *Cuphea*). During the dry season, when there was a greater diversity of floral resources, small‐billed migratory hummingbirds visited *R. ciliatum* flowers at high elevations, while resident and migrant hummingbirds with longer bills segregated across diverse floral patches at low‐ and mid‐elevation forests. When floral resources are abundant, hummingbirds tend to prefer morphologically matched resources over abundant ones, highlighting the importance of feeding efficiency provided by matching floral resources (Maglianesi et al. 2014; Vitória, Vizentin‐Bugoni, and Duarte 2018; Sonne et al. 2019). This supports the idea that trait matching and niche partitioning among hummingbirds are potential mechanisms promoting coexistence in diverse communities (Stiles 1978; Maglianesi et al. 2015). Furthermore, morphology and phenology can act as forbidden links, namely, interactions restricted due to physical or temporal decoupling between species (Jordano, Bascompte, and Olesen 2003), playing a greater role than abundance in structuring ecological networks (Vizentin‐Bugoni, Maruyama, and Sazima 2014).

#### 4.4.2 Phylogenetic Signal

Phylogenetic relatedness may influence interaction frequencies in pollination networks, as closely related species are expected to interact more frequently with the same or related partners (Rezende, Jordano, and Bascompte 2007; Graham et al. 2012). Our results did not support this prediction since interaction frequencies and partner taxonomic identity were independent of phylogenetic relatedness in most elevation and season networks. This was the case for plants, where closely related species did not share similar interaction frequencies or the same hummingbird partners. Instead, non‐phylogenetic factors, have a greater influence on plant–pollinator interactions (Wolowski, Carvalheiro, and Freitas 2017). Moreover, floral variation in our study system spans a relatively limited set of phenotypes, including cases of convergent evolution of floral traits adapted to hummingbird pollination (ornithophilous flowers; Faegri and van der Pijl 1979). Thus, closely related plant species would not necessarily be more similar or more likely to attract the same pollinator species.

Results were similar for interactions established by hummingbirds in low‐ and mid‐elevation forests and during the dry season, where interaction frequencies or feeding preferences were not more similar in closely related hummingbird species than expected by chance. However, for the smaller high‐elevation network (where plant diversity was lower), a significant co‐phylogenetic effect suggests the use of closely related plant species by closely related hummingbird species. Similarly, during the rainy season (when floral abundance was lower), some closely related species of hummingbirds were more likely to visit the same plant species (e.g., *S. platycercus* and *S. rufus* primarily feeding on *S. purpurea*). This pattern has been documented in the Andean mountains, and it has been attributed to a greater influence of competition at mid elevations and environmental filtering at high elevations (Graham et al. 2009), where more closely related species could have similar tolerances to extreme abiotic conditions. In our study, lower‐ and mid‐elevation forests (pine‐oak and fir) offer resources for hummingbirds from different clades. In contrast, the higher subalpine environment offers an abundant resource for several migrant species, including three *Selasphorus* species, and resident hummingbirds, two of which belong to the same clade (*E. fulgens* and *L. amethystinus*) and use the same plant species. Similarly, two species of plants in the genus *Lupinus* are visited by the core hummingbird species *B. leucotis*. Overall, the structure of plant–hummingbird interaction networks suggests that phenological and morphological matching among plant and hummingbird species has more influence than evolutionary factors in assembling plant and hummingbird communities (Vitória, Vizentin‐Bugoni, and Duarte 2018).

## 5 Conclusions

The species richness and diversity of blooming plants and hummingbirds were similar between the lower‐elevation pine‐oak and the mid‐elevation fir forests of Nevado de Colima, but a pronounced decline of blooming plant diversity was observed in the subalpine forest. High beta diversity metrics suggest that plants, being sessile organisms, adapt to specific thermal conditions along the elevational gradient, while hummingbirds are better able to move across elevations and forest types. Network specialization was inversely related to species richness in the subalpine forest, showing the influence of specific plant and hummingbird species that establish asymmetrical specialized interactions. Similarly, network specialization was highest during the rainy season, the period of lowest floral abundance, suggesting greater resource partitioning among hummingbird species. Beta diversity of interactions along the elevational gradient was high, primarily driven by the observed species turnover in the plant community. In contrast, seasonal variation was more strongly influenced by interaction rewiring, driven by the seasonal movements of hummingbird species. Interaction frequencies were strongly influenced by the phenological overlap between blooming plants and hummingbird species, where migratory hummingbirds are essential components of temporal variation. Morphological matching was an important driver of interaction frequencies only at the lower‐elevation forest (pine‐oak) and during the dry season, when flowering plant and hummingbird communities were more diverse. Phylogenetic relatedness had a minimal influence on the structuring of most pollination networks and was significant for hummingbirds only in the subalpine forest and during the rainy season, when a co‐phylogenetic effect was observed. These results underscore the significance of spatiotemporal dynamics in structuring pollination networks, driven by elevational and seasonal fluctuations in floral resource availability and by the elevational and latitudinal movements of hummingbirds. Our results highlight the critical role of high‐mountain ecosystems in providing year‐round floral resources for hummingbirds, especially in a regional context where lowland ecosystems are dry, seasonal, and subject to high levels of habitat disturbance.

## Author Contributions

**Eugenia M. Sentíes‐Aguilar:** conceptualization (lead), data curation (equal), formal analysis (equal), investigation (lead), methodology (equal), validation (lead), visualization (lead), writing – original draft (lead), writing – review and editing (lead). **Silvana Martén‐Rodríguez:** conceptualization (lead), funding acquisition (equal), investigation (equal), methodology (equal), project administration (lead), resources (lead), supervision (equal), writing – original draft (lead), writing – review and editing (lead). **Guillermo Huerta‐Ramos:** formal analysis (equal), investigation (supporting), software (equal), writing – original draft (supporting), writing – review and editing (supporting). **Sergio Díaz‐Infante:** investigation (equal), methodology (supporting), writing – review and editing (equal). **Gabriel López‐Segoviano:** formal analysis (supporting), investigation (equal), writing – review and editing (equal). **Armando Aguirre‐Jaimes:** methodology (equal), supervision (equal), writing – review and editing (equal). **Mauricio Quesada‐Avendaño:** investigation (supporting), project administration (supporting), resources (equal), writing – review and editing (equal). **Jorge Cortés‐Flores:** formal analysis (equal), software (equal), validation (supporting), writing – review and editing (equal). **María del Coro Arizmendi:** methodology (equal), supervision (equal), writing – review and editing (equal).

## Conflicts of Interest

The authors declare no conflicts of interest.

## Supporting information


Appendix S1–S7.

## Data Availability

The data that support the findings of this study are available in Zenodo Data at https://zenodo.org/records/13755893 (http://doi.org/10.5281/zenodo.13755893).
