# Supplementary material for: Elevational and Seasonal Patterns of Plant–Hummingbird Interactions in a High Tropical Mountain
Source: Ecol Evol. 2024 Oct 24;14(10):e70469. doi: 10.1002/ece3.70469 (PMC11500213; doi:10.1002/ece3.70469)
Supplement: Supplementary file 1 — Appendix S1–S7. [file ECE3-14-e70469-s001.docx]

**APPENDIX S1. Specimen information for plant species recorded in plant-hummingbird interactions at Nevado de Colima Volcano National Park, México. The Voucher specimen ID, collection number, and herbarium code are listed for each species.**

| **Family** | **Species** | **ID Voucher specimen** | **Collection number** | **Herbarium ID** |
| --- | --- | --- | --- | --- |
| Asteraceae | *Cirsium ehrenbergii* | 272670 | ESA5 | IEB |
|  | *Cirsium jaliscoense* | 272683 | NYG19 | IEB |
|  | *Dahlia tenuicaulis* | 272667 | GHR170 | IEB |
|  | *Roldana angulifolia* | 272664 | LC5 | IEB |
|  | *Senecio callosus* | 272662 | ESA24 | IEB |
| Bromeliaceae | *Tillandsia bourgaei* | 272661 | LC12 | IEB |
| Campanulaceae | *Lobelia laxiflora* | 215844 | ESA07 | IBUG |
| Ericaceae | *Arbutus xalapensis* | 215769 | GHR307 | IBUG |
| Fabaceae | *Lupinus montanus* | 215765 | GHR136 | IBUG |
|  | *Lupinus reflexus* | 215761 | KDPA13 | IBUG |
| Grossulariaceae | *Ribes ciliatum* | 215756 | GHR149 | IBUG |
| Lamiaceae | *Salvia elegans* | 272656 | GHR321 | IEB |
|  | *Salvia gesneriiflora* | 272652 | MP5 | IEB |
|  | *Salvia iodantha* | 272653 | GHR329 | IEB |
|  | *Salvia longistyla* | 272650 | GHR311 | IEB |
|  | *Salvia mexicana* | 272649 | GHR317 | IEB |
|  | *Salvia microphylla* | 272647 | LC8 | IEB |
|  | *Salvia purpurea* | 272659 | KDPA2 | IEB |
|  | *Salvia ramamoorthyana* | 272657 | GHR372 | IEB |
|  | *Stachys pilosissima* | 272644 | ESA27 | IEB |
| Lythraceae | *Cuphea watsoniana* | 215750 | ESA19 | IBUG |
| Malvaceae | *Tilia americana* | 215749 | GHR200 | IBUG |
| Onagraceae | *Fuchsia cylindracea* | 272642 | GHR271 | IEB |
|  | *Fuchsia microphylla* | 272640 | GHR270 | IEB |
| Orobanchaceae | *Castilleja cryptandra* | 215722 | ESA29 | IBUG |
|  | *Castilleja tenuiflora* | 215725 | GHR208 | IBUG |
|  | *Lamourouxia macrantha* | 215724 | GHR265 | IBUG |
|  | *Lamourouxia xalapensis* | 215723 | GHR322 | IBUG |
| Plantaginaceae | *Penstemon roseus* | 215710 | ESA30 | IBUG |
| Solanaceae | *Cestrum laxum* | 215378 | GHR278 | IBUG |
|  | *Cestrum thyrsoideum* | 215377 | GHR304 | IBUG |

ID Voucher specimen corresponds to the key number assigned in the herbariums, and Collection number is the code assigned when the specimen by its collector. Herbarium ID: The “Herbarium Luz María Villarreal de Puga” (IBUG) of the Universidad de Guadalajara, and the “Herbarium Graciela Calderón y Jerzy Rzedowsky” (IEB) of the Instituto de Ecología, A.C.

**APPENDIX S2. Insect groups observed visiting the flowers of plant species used by hummingbirds in the Nevado de Colima Volcano National Park, México.**

| **Plant species** | **Insect groups** | | | | | | |
| --- | --- | --- | --- | --- | --- | --- | --- |
|  | **Bees** | **Bumblebees** | **Flies** | **Butterflies** | **Moths** | **Beetles** | **Passerine bird** |
| *Arbutus xalapensis* | x |  | x |  |  |  |  |
| *Castilleja cryptandra* | x |  | x | x |  |  |  |
| *Castilleja tenuiflora* | x |  |  | x |  |  |  |
| *Cestrum laxum* |  |  |  |  |  |  |  |
| *Cestrum thyrsoideum* |  |  |  | x |  |  |  |
| *Cirsium ehrenbergii* | x | x | x | x | x | x |  |
| *Cirsium jaliscoense* | x | x |  |  |  | x |  |
| *Cuphea watsoniana* |  |  | x | x |  |  |  |
| *Dahlia tenuicaulis* | x | x | x | x |  |  |  |
| *Fuchsia cylindracea* | x | x |  | x |  |  |  |
| *Fuchsia microphylla* |  | x | x | x |  |  |  |
| *Lobelia laxiflora* | x | x | x | x |  |  |  |
| *Lupinus montanus* |  | x | x |  |  |  |  |
| *Lupinus reflexus* |  | x | x |  |  |  |  |
| *Penstemon roseus* | x | x |  |  |  |  |  |
| *Ribes ciliatum* | x | x |  | x | x |  | x |
| *Roldana angulifolia* |  | x |  |  |  |  |  |
| *Salvia elegans* |  | x | x | x |  |  |  |
| *Salvia gesneriiflora* |  |  | x |  |  | x |  |
| *Salvia iodantha* | x | x | x | x |  | x |  |
| *Salvia longistyla* | x | x |  | x |  |  |  |
| *Salvia mexicana* | x | x | x |  | x | x |  |
| *Salvia microphylla* |  | x |  |  |  |  |  |
| *Salvia purpurea* | x | x |  | x | x |  |  |
| *Salvia ramamoorthyana* | x | x | x |  |  |  |  |
| *Salvia* sp. |  |  |  | x |  |  |  |
| *Senecio callosus* |  | x |  |  |  |  |  |
| *Stachys pilosissima* | x | x | x | x |  |  |  |
| *Tilia americana* |  | x |  |  | x |  |  |
| *Tillandsia bourgaei* |  |  |  |  |  |  |  |

**APPENDIX S3. Plant species used as floral resources by hummingbirds across elevations and seasons at Nevado de Colima Volcano National Park, México.**

| **Family** | **Plant species** | **Elevation gradient** | | | **Season** | |
| --- | --- | --- | --- | --- | --- | --- |
|  |  | **Low** | **Mid** | **High** | **Dry** | **Rainy** |
| Asteraceae | *Cirsium ehrenbergii* |  | x | x | x | x |
|  | *Cirsium jaliscoense* | x | x |  | x | x |
|  | *Dahlia tenuicaulis **^a^ (Pr) | x | x |  | x | x |
|  | *Roldana angulifolia* | x | x | x | x |  |
|  | *Senecio callosus* |  | x | x | x | x |
| Bromeliaceae | *Tillandsia bourgaei* | x |  |  | x |  |
| Campanulaceae | *Lobelia laxiflora* | x | x |  | x | x |
| Ericaceae | *Arbutus xalapensis* | x | x |  | x |  |
| Fabaceae | *Lupinus montanus* |  |  | x | x | x |
|  | *Lupinus reflexus* (EN) |  |  | x | x | x |
| Grossulariaceae | *Ribes ciliatum* |  | x | x | x |  |
| Lamiaceae | *Salvia elegans* | x | x |  | x | x |
|  | *Salvia gesneriiflora* | x | x |  | x | x |
|  | *Salvia iodantha* | x | x |  | x | x |
|  | *Salvia longistyla* |  | x |  | x | x |
|  | *Salvia mexicana* | x | x |  | x | x |
|  | *Salvia microphylla* |  | x |  | x | x |
|  | *Salvia purpurea* | x | x |  | x | x |
|  | *Salvia ramamoorthyana* | x | x |  | x | x |
|  | *Salvia* sp. |  | x |  | x | x |
|  | *Stachys pilosissima* | x | x |  | x | x |
| Lythraceae | *Cuphea watsoniana* | x | x |  | x | x |
| Malvaceae | *Tilia americana* var. m*exicana* *^a^ (P) | x |  |  |  | x |
| Onagraceae | *Fuchsia cylindracea* | x | x |  | x | x |
|  | *Fuchsia microphylla* | x | x |  | x | x |
| Orobanchaceae | *Castilleja cryptandra *** |  | x | x | x | x |
|  | *Castilleja tenuiflora* | x | x |  | x | x |
|  | *Lamourouxia macrantha* |  | x |  |  | x |
|  | *Lamourouxia xalapensis* | x | x |  | x | x |
| Plantaginaceae | *Penstemon roseus* *^b^ | x | x | x | x | x |
| Solanaceae | *Cestrum laxum* |  | x |  | x |  |
|  | *Cestrum thyrsoideum* | x |  |  | x |  |

Elevation gradient: Low (2400-2600 m a.s.l.), Mid (2800-3100 m), High (3400-3700 m). Seasons: Dry (November, January, March), Rainy (May, July, September). Mexican endemic species (*^a^ NOM-059-SEMARNAT-2010, 2019; *^b^ Lara & Ornelas, 2008). Endemic species to the Nevado de Colima (** Nesom, 1992). Protected species: extinction endangered species (P), special protection species (Pr; NOM-059-SEMARNAT-2010, 2019); Endangered species (EN; IUCN, 2024).

**APPENDIX S4. Hummingbird clades and species (Trochilidae) recorded across elevations and seasons at Nevado de Colima Volcano National Park, México.**

| **Clade** | **Hummingbird species** | **English name** | **Seasonality** | **Elevation gradient** | | | **Season** | |
| --- | --- | --- | --- | --- | --- | --- | --- | --- |
|  |  |  |  | **Low** | **Mid** | **High** | **Dry** | **Rainy** |
| Mangoes | *Colibri thalassinus* | Mexican Violetear | EM | x | x | x | x | x |
| Mountain Gems | *Eugenes fulgens* | Rivoli's Hummingbird | R | x | x | x | x | x |
|  | *Lampornis amethystinus* | Amethyst-throated Mountain-gem | R | x | x | x | x | x |
|  | *Lampornis clemenciae* | Blue-throated Mountain-gem | EM | x | x |  | x | x |
| Bees | *Tilmatura dupontii* (A) | Sparkling-tailed Hummingbird | EM |  | x |  | x |  |
|  | *Archilochus colubris* | Ruby-throated Hummingbird | LM |  | x | x | x |  |
|  | *Calypte costae* | Costa's Hummingbird | LM | x |  |  | x |  |
|  | *Selasphorus calliope* | Calliope Hummingbird | LM | x |  | x | x | x |
|  | *Selasphorus rufus* (NT) | Rufous Hummingbird | LM | x | x | x | x | x |
|  | *Selasphorus platycercus* | Broad-tailed Hummingbird | LM | x | x | x | x | x |
|  | *Selasphorus heloisa* * | Bumblebee Hummingbird | EM | x | x |  |  | x |
| Emeralds | *Basilinna leucotis* | White-eared Hummingbird | R | x | x | x | x | x |
|  | *Ramosomyia violiceps* | Violet-crowned Hummingbird | EM | x | x |  | x | x |
|  | *Saucerottia beryllina* | Berylline Hummingbird | EM | x | x | x | x | x |

Hummingbird clades are according to McGuire et al. (2014), and the taxonomic order of the species corresponds to Chesser et al. (2023). Elevation gradient: Low (2400-2600 m a.s.l.), Mid (2800-3100 m), High (3400-3700 m). Seasons: Dry (November, January, March), Rainy (May, July, September). Hummingbird seasonality: Resident (R), Latitudinal migratory (LM), Elevational migrant (EM). Mexican endemic species (* NOM-059-SEMARNAT-2010, 2019). Protected species: Threatened species (A; NOM-059-SEMARNAT-2010, 2019). Near Threatened species (NT; IUCN, 2024).

**APPENDIX S5. Alpha diversity of blooming plant and hummingbird species across the elevation gradient in the Nevado de Colima Volcano National Park, México.**


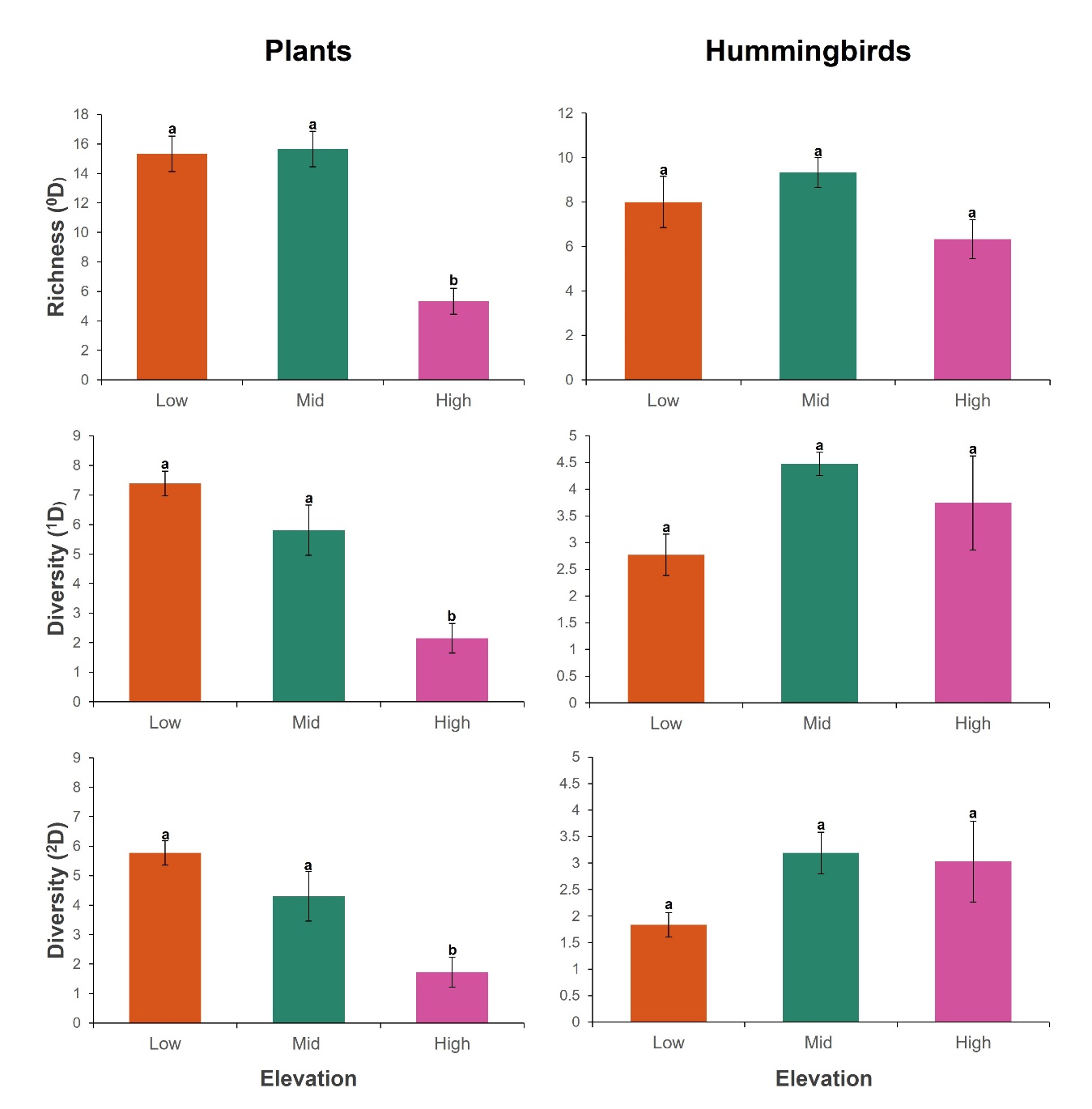


Hill´s numbers for alpha diversity: ^0^D: Richness observed; ^1^D: Shannon diversity (abundance); ^2^D: Simpson's diversity (dominant species). Different superscripts show statistical differences between sites across the elevation gradient (Tukey HSD test).

**APPENDIX S6. NMDS analysis of the community composition of blooming plant and hummingbird species recorded at Nevado de Colima Volcano National Park, México.**


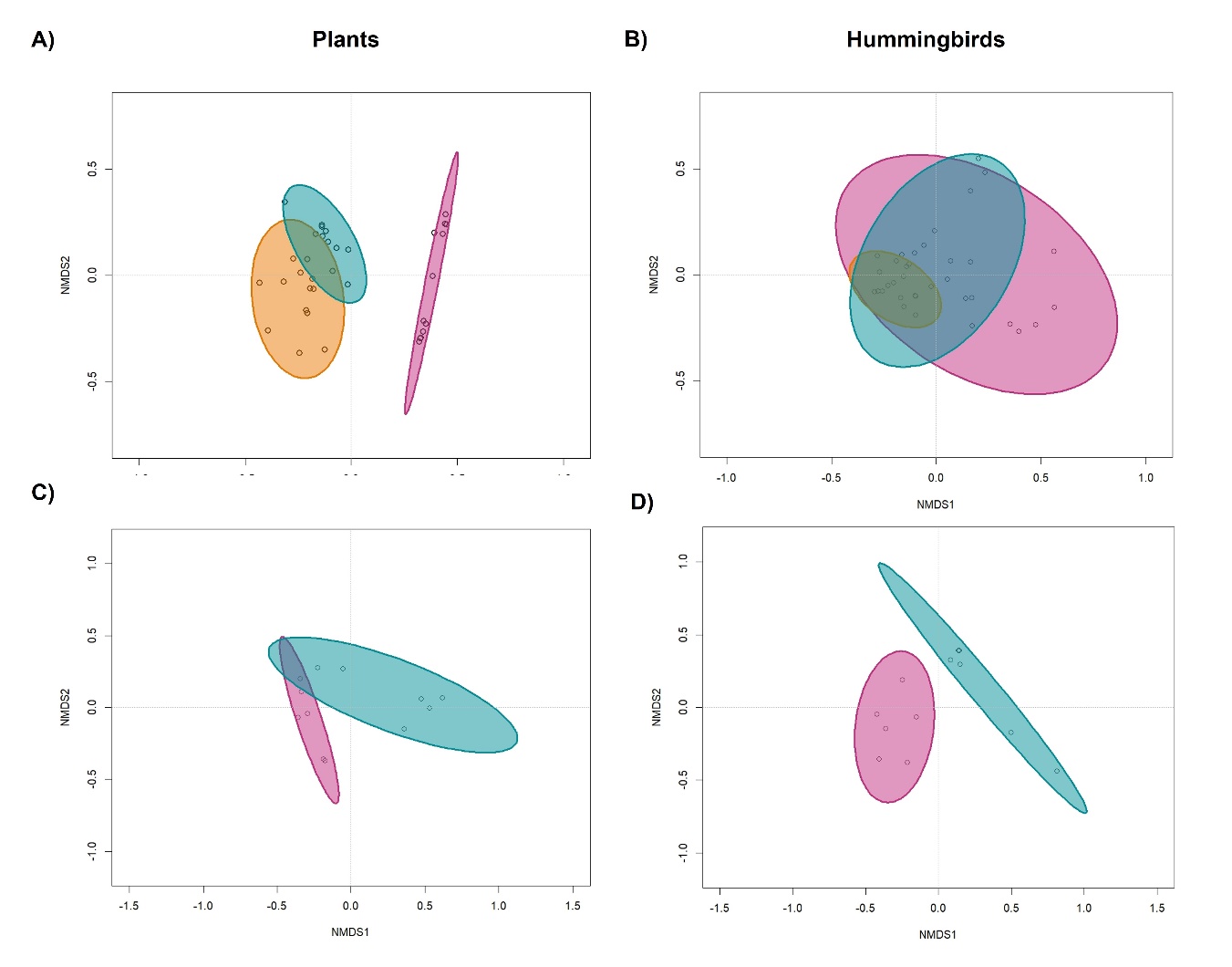


Elevational gradient: (A) blooming plants, (B) hummingbirds. Seasons: C) plants, (D) hummingbirds. Elevation gradient: low site (orange), mid site (aquamarine), high site (purple). Season: dry (purple), rainy (aquamarine). For plants, the distance–decay pattern is observed. Ellipsoids area corresponding 95% confidence interval.

**APPENDIX S7. Blooming plant and hummingbird species phenologies across six samplings conducted over two years in the Nevado de Colima Volcano National Park, México. Dry season: (November, January, March); Rainy season (May, July, September).**

**
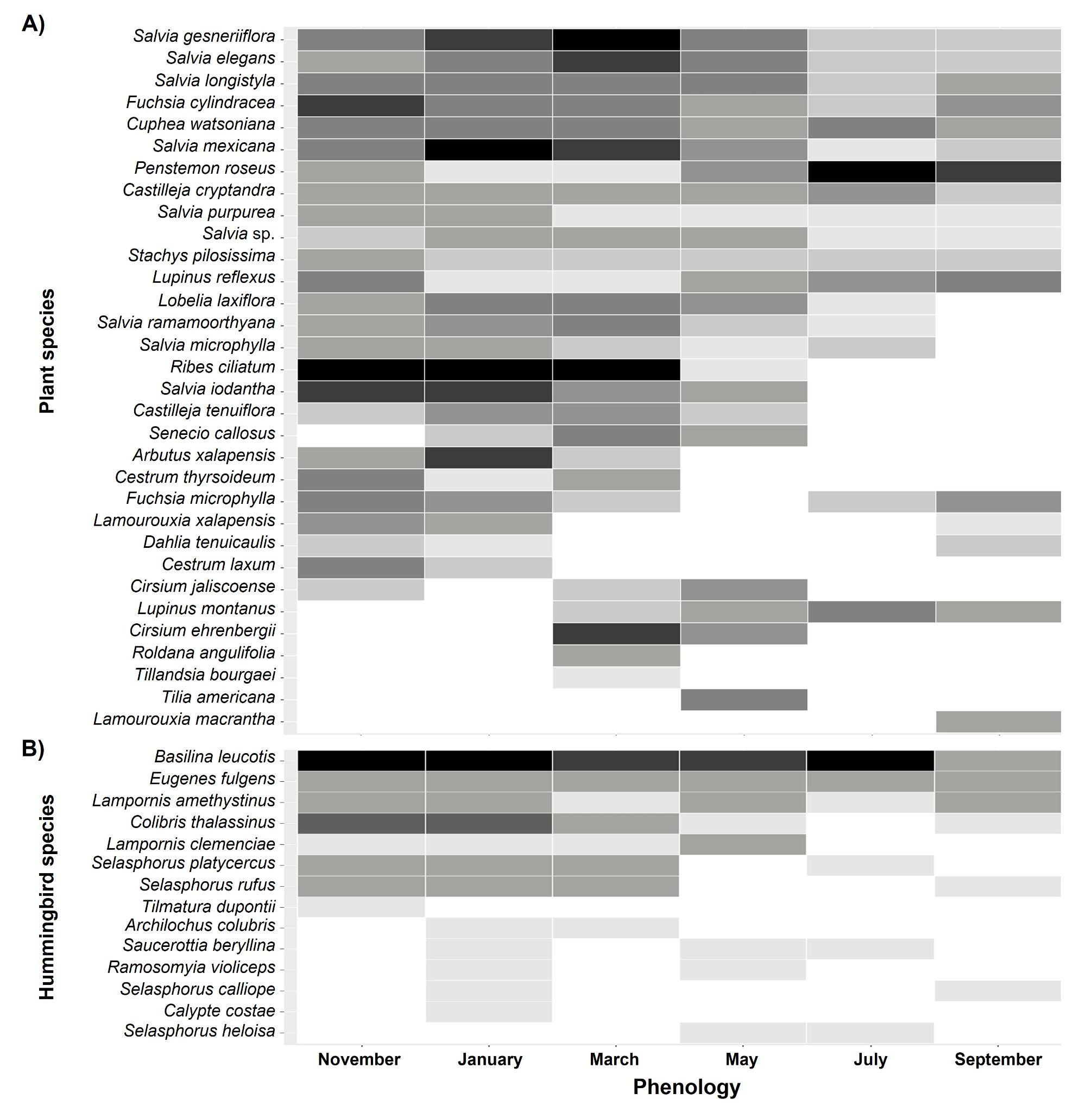
**

The increase in squares' color intensity indicates greater (A) floral or (B) hummingbird abundance.

**Appendices References**

Chesser RT, Billerman SM, Burns KJ, Cicero C, Dunn JL, Hernández-Baños BE, et al. Check-list of North American Birds (online). American Ornithological Society. 2023. <https://checklist.americanornithology.org/taxa/>

Lara C, Ornelas JF. Pollination ecology of *Penstemon roseus* (Plantaginaceae), an endemic perennial shifted toward hummingbird specialization?. Plant Syst. Evol. 2008; 271:223-237. <https://doi.org/10.1007/s00606-007-0624-0>

McGuire JA, Witt CC, Remsen JV, Corl A, Rabosky DL, Altshuler DL, Dudley R. Molecular phylogenetics and the diversification of hummingbirds. Curr. Biol. 2014; 24(8):910-916. <https://doi.org/10.1016/j.cub.2014.03.016>

Nesom GL. Castilleja section Euchroma (Scrophulariaceae) in Mexico: new species and comments on other taxa. Phytologia. 1992; 73(5):384-388.

NOM-059-SEMARNAT-2010. Norma Oficial Mexicana (NOM). Official Mexican standard for the environmental protection of Mexican species of wild flora and fauna. 2019. <http://legismex.mty.itesm.mx/normas/ecol/semarnat059-ModAnexoIII2019_11.pdf>

IUCN. International Union for Conservation of Nature. The IUCN Red List of Threatened Species. 2024. Version 2024-1. <https://www.iucnredlist.org>.
